# Coherent Raman Imaging of Live Muscle Sarcomeres Assisted by SFG Microscopy

**DOI:** 10.1038/s41598-017-09571-w

**Published:** 2017-08-23

**Authors:** Hyunmin Kim, Do-Young Kim, Kyung-Il Joo, Jung-Hye Kim, Soon Moon Jeong, Eun Seong Lee, Jeong-Hoon Hahm, Kyuhyung Kim, Dae Woon Moon

**Affiliations:** 1 Companion Diagnostics and Medical Technology Research Group, DGIST, Daegu, 42988 Republic of Korea; 2 Department of Brain and Cognitive Science, DGIST, Daegu, 42988 Republic of Korea; 3 School of Electronics Engineering, Kyungpook National University, Daegu, 41566 Republic of Korea; 4 Smart Textile Convergence Research Group, DGIST, Daegu, 42988 Republic of Korea; 5 Center for Nanometrology, Korea Research Institute of Standards and Science, 267 Gajeong-ro, Yuseong-gu, Daejeon, 34113 Republic of Korea; 6 Center for Plant Aging Research, Institute for Basic Science, Daegu, 42988 Republic of Korea; 7 Department of New Biology, DGIST, Daegu, 42988 Republic of Korea

## Abstract

In this study, we used spectrally focused coherent anti-Stokes Raman scattering (spCARS) microscopy assisted by sum-frequency generation (SFG) to monitor the variations in the structural morphology and molecular vibrations of a live muscle of *Caenorhabditis elegans*. The subunits of the muscle sarcomeres, such as the M-line, myosin, dense body, and α-actinin, were alternatively observed using spCARS microscopy for different sample orientations, with the guidance of a myosin positional marker captured by SFG microscopy. Interestingly enough, the beam polarization dependence of the spCARS contrasts for two parallel subunits (dense body and myosin) showed a ~90° phase difference. The chemically sensitive spCARS spectra induced by the time-varying overlap of two pulses allowed (after a robust subtraction of the non-resonant background using a modified Kramers–Krönig transformation method) high-fidelity detection of various genetically modified muscle sarcomeres tuned to the C-H vibration (2800–3100 cm^−1^). Conversely, SFG image mapping assisted by phase-retrieved spCARS spectra also facilitated label-free monitoring of the changes in the muscle content of *C. elegans* that are associated with aging, based on the hypothesis that the C-H vibrational modes could serve as qualitative chemical markers sensitive to the amount and/or structural modulation of the muscle.

## Introduction

Coherent Raman scattering (CRS) shows a selectively enhanced efficiency for the molecular resonance induced by a phase-matched multiple photon excitation tuned to the specific vibrational mode. Thereby, this technique has matured as a real-time chemically sensitive imaging^1^ method in materials science; a critical example of this is the imaging of single molecular breathing^2^ contributed by the effect of SECARS (surface-enhanced coherent anti-Stokes Raman scattering^3^, which might be a multi-photon-excitation mode SERS (surface-enhanced Raman scattering). Also, the importance of CRS should be stressed even more in the design of highly disordered materials, where the optical coherency of Raman scattering would be highly influenced in a backward direction^4^.

*Caenorhabditis elegans* (*C. elegans*) has played a central role in the investigation of numerous problems in biological science due to its optical transparency, moderate size (~1 mm long), short lifespan (~3 weeks), and easy maintenance using standard *Escherichia coli* as a food source, and it offers superb *in vivo* experimental accessibility^5–8^. In particular, *C. elegans* muscle has been used as an endogenous biomarker of different biological processes, including aging. Although the morphological changes of the muscle can be identified using polarized light microscopy^9^ combined with immunofluorescence assays^10^, the shortcomings of this method (it requires sample fixation, lacks the ability for molecular identification, and has low 3D resolving power) led researchers to adopt confocal fluorescence microscopy^11^ instead. Sum-frequency generation (SFG) and second-harmonic generation (SHG) are nonlinear optical processes sensitive to structurally oriented biomolecules—such as collagen, microtubules, muscles, and starch—and that may serve to visualize the muscle fibrils of nematodes noninvasively^12^. A brief history of biological SHG microscopy would begin with its first use in cell membranes^13^, followed by imaging of microtubule assemblies in brain tissue^14^ and an *in vivo* study of muscle dynamics in humans^15^.

Spectrally focused coherent anti-Stokes Raman scattering microscopy (spCARS) has also been extensively investigated as a method to extract chemical information from bioanalytical specimens. This kind of microscopy involves overlapping two or more chirped femtosecond pulses and has been advantageous mostly due to its fast signal collection and smooth wavelength variation induced by time delay. The spCARS has been used to study microcrystals^16^, polymer beads^17^, live cells, or animals^18–20^. In fact, CARS microscopy will be one of the strongest candidates to overcome the limitations of SHG microscopy in muscular imaging, should it be able to visualize sarcomere filaments. Furthermore, the inherent simultaneous generation of nonlinear optical signals such as SHG and SFG, and also spCARS, that takes place when mixing spectrally focused femtosecond pulses implies that the advantages of each signal can be synergistically combined.

Taking into account this background and the need for chemically sensitive and high-spatial-resolution nonlinear optical imaging of biological samples, we used spCARS microspectroscopy generated by the overlap of two optically chirped ultrafast femtosecond (~120 fs) pulses, combined with SFG microscopy, to study anaesthetized *C. elegans* and critically evaluate the morphological and chemical changes induced by genetic modification and aging on its muscle sarcomeres.

## Materials and Methods

### Strains

The *C. elegans* N2 Bristol strain was used as the wild type (WT). We also used *unc-89*(e1460), PD4251 ccIs4251 I, *dpy-20*(e1282) IV, RW1596 *myo-3*(st386) V, and stEx30. All the strains were obtained from the Caenorhabditis Genetic Center and were maintained on nematode growth medium (NGM) plates with OP50 at 20 °C.

### Sample preparation

*C. elegans* samples were mounted on a 2% agarose pad and placed in a 100 mM sodium azide solution inside a round-shaped glass-bottomed dish, in order to facilitate the adjustment of the sample orientation. Then, the sample-containing gels were covered with a coverslip and sealed with transparent nail polish to secure their position for imaging.

### Aged sample preparation

To synchronize the growth of the worms, we placed adult specimens on an NGM plate and allowed them to lay eggs for 3 hours. Then, the adult worms were removed and the progenies were cultured until they reached the optimal age for the experiments^21^.

## Results and Discussion

**Figure 1:**
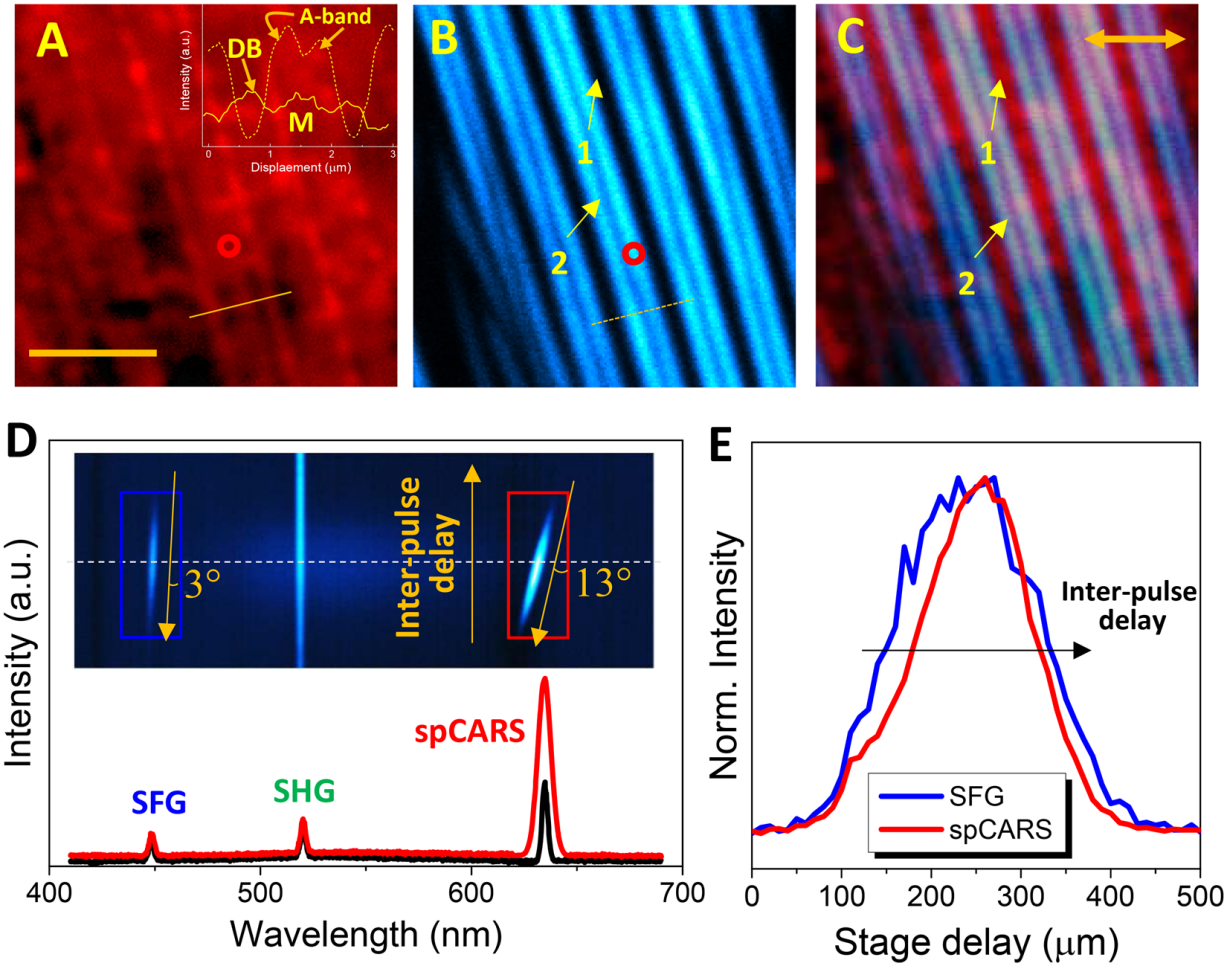
Spectrally focused CARS (spCARS) microspectroscopy assisted by SFG for C. elegans muscle subunits. Body wall muscle of WT *C. elegans* visualized using the **(A**) spCARS and **(B)** SFG techniques. The scale bar in (**A**) represents 5 μm, and the inset compares the line profiles along the solid and dotted lines in (**A**) and (**B**). (DB: dense body; M: M-line.) **(C)** Overlay image of (**A**) and (**B**). The two-headed arrow indicates the polarization direction of the two beams (pump and Stokes) and a polarizer located right before the detector (notice that the three are parallel). (**D)** Wavelength spectra of WT *C. elegans* muscle (corresponding to the red circles in Fig. 1a, b and also to the white dashed line in the inset spectrogram) when the time delay is zero, in the presence (black) and absence (red) of a chirping glass. **(E)** SFG/spCARS spectra obtained from the blue (SFG) and red (spCARS) rectangles in (**D**).

Figure 1A,B show microscopic images of the *C. elegans* muscle obtained through the spCARS and SFG techniques, respectively. Our SFG (452 nm) signals, imaged at the time when the pump and Stokes pulses are overlapped, played the role of a “position ruler” for CARS imaging. This had the advantage of simultaneously avoiding autofluorescence (compared to SHG at 520 nm) and reinforcing the deep-tissue-scanning capabilities (compared to SHG at 400 nm). A previous study with an *ex vivo* sample^22^ showed that skeletal muscle could be fully characterized using CARS (visualizing the Z-line, actomyosin, actin, and myosin), whereas the only muscle subunit seen with SHG was myosin. Similarly, Fig. 1A shows noticeable periodic bright regions (1) associated with the dense body (DB, a homolog of the Z-line) as well as weak evidence for the M-line (red circle). We were also able to distinguish the DB from a globular-shaped α-actinin embodied in it. Unless otherwise noticed, all spCARS images in this work were obtained using a ~8 mW pump beam (802 nm) and a ~30 mW Stokes beam (1041 nm) for a convenient comparison with the above literature at 2860 cm^−1^. On the other hand, in Fig. 1B, SFG allows us to clearly appreciate the A-band^23^ (2) of the myosin fiber, which does not overlap with (1), as shown in the overlay image of Fig. 1C. The inset in Fig. 1A identifies the positions of each subunit (M-line, DB, and A-band) along the line intensity profiles corresponding to the solid line in (a) and the dotted line in (b). However, in contrast to the *ex vivo* experiment described above, the actomyosin areas were not clearly resolved by spCARS. Figure 1D shows the spectrum of the muscle of a *C. elegans* worm in the presence of a chirping glass (black) or without it (red), using a pump beam wavelength of 792 nm. We see that the chirping glass shifts the FWHM of the spCARS spectrum from ~6 to ~3 nm. The inset shows the SFG, SHG (by the Stokes beam), and spCARS time-wavelength spectrograms as a function of the interpulse delay. The broadband (450–600 nm) background induced by the autofluorescence of the animals is clearly observed. Also, note the ~4-fold difference between the spCARS (13°) and SFG (3°) light stretching (λ/ΔT, i.e. wavelength/inter-pulse delay). Figure 1E shows the spCARS (red) and SFG (blue) spectra collected at a fixed focal point (0.3–0.4 μm laterally; <0.6–0.8 μm axially) over the target area (the red circle in (a) and (b)) by a CCD camera, clearly demonstrating the difference in the signal-to-noise ratio (S/N) and shape of the spectral signals.

**Figure 2:**
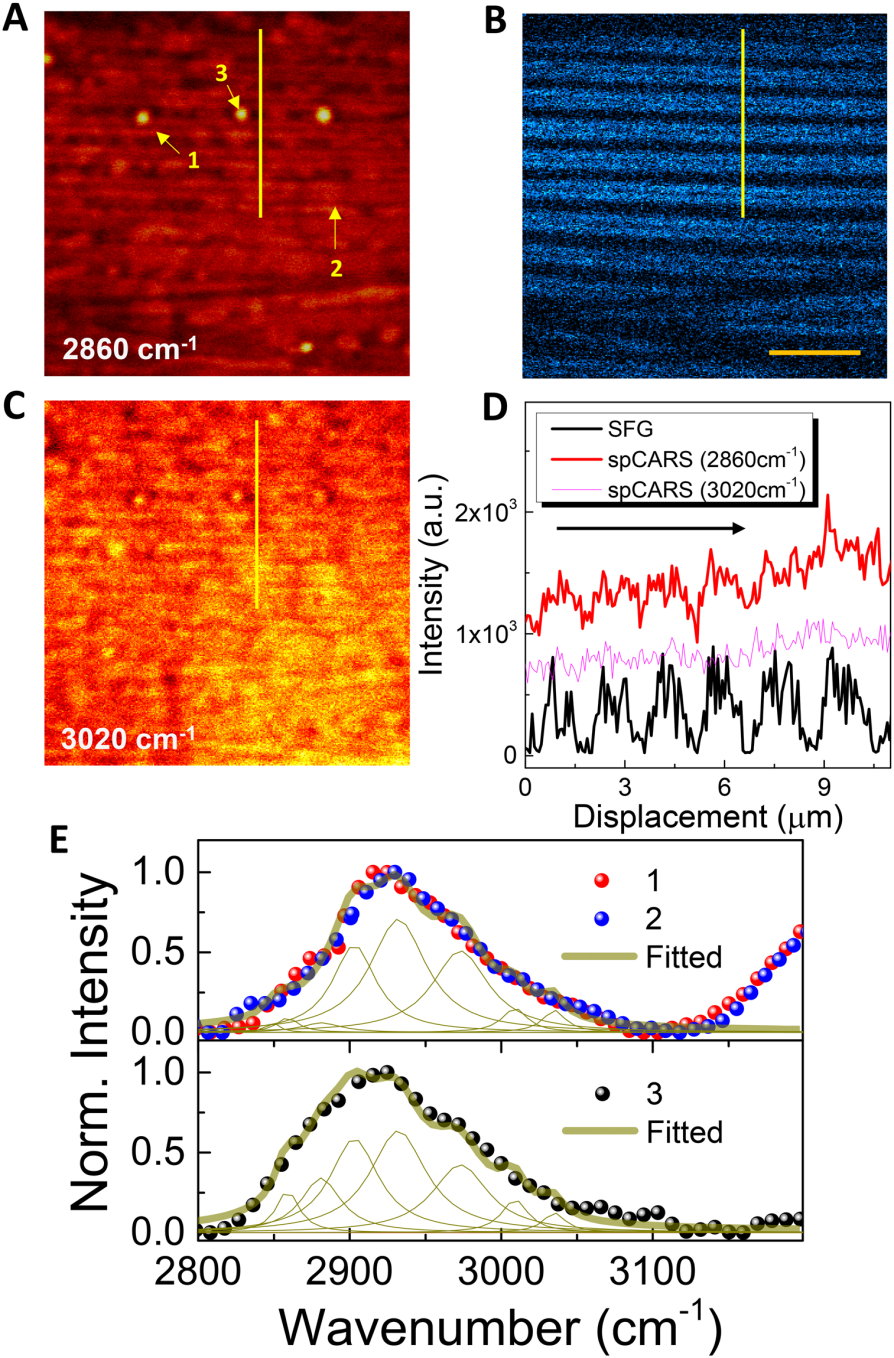
Chemically sensitive identification of muscle subunits. (**A**) The spCARS and (B) SFG images of body-wall muscle near the vulva of WT *C. elegans* for the 2860 cm^−1^ vibration mode; (**C**) spCARS image for 3020 cm^−1^. The scale bar in (**B**) represents 10 μm. **(D)** Line profile along the solid line in (**A**) and (B). (**E**) Normalized phase-retrieved spCARS spectra for 1–3 in (**A**).

Figure 2 shows an example in which the myosin A-bands were clearly resolved by spCARS microspectroscopy. It is remarkable that such a “myosin-rich” case was mainly seen when the myosin fiber was at a 40–60° angle with respect to the beam polarization (horizontal), as demonstrated in Fig. S3. The spCARS image of two parallel A-band myosin structures was clearer when the pump wavelength was set to 2860 cm^−1^ (a) than for 3020 cm^−1^ (c); Fig. 2B shows an SFG image of the same region. As the ideal polarization condition (~45°) was not met, the SFG signal was weak, but still served to confirm the position of the myosin found by spCARS through the comparison of the line profiles of both signals (Fig. 2D). The phase-retrieved spCARS spectra in Fig. 2E correspond to various positions in the images, suspected to be myosin (1), the dense body (DB) (2), and a lipid droplet (3). See the supporting information for the details of the phase-retrieval process, based on a modified Kramers-Krönig (KK) transformation method^24^. To gain a more quantitative and intuitive insight, we performed Raman peak separation in the highly congested region of 2800–3000 cm^−1^ using the vibrational modes available in the literature^25^. The results are summarized in Table S1, in the supporting information. The difference between the spCARS spectral profiles of the myosin and the DB structures was hardly noticeable, while that of the lipid droplet was characterized by the large area (>5 times larger than for myosin and the DB) of the peak corresponding to the 2881 cm^−1^ vibration mode. This large area can be understood in terms of the triggering of the Fermi resonance, in accordance with the periodic lipid configuration^26^.

The polarization dependence of the SFG signals on the sample orientation was also tested. To this end, we used the laboratory coordinates defined in Fig. S7A, where θ is the angle between the sample and the excitation beam, which is varied in the SFG images shown in the upper (when the polarization of the analyzer is parallel to the pump and Stokes beams, xxx) and lower (when the polarization of the analyzer is perpendicular to the pump and Stokes beams, xxy) panels of Fig. S7B. We can clearly see that the SFG intensity from the body wall muscle of *C. elegans* is maximum for different values of θ, namely 45° and 90° for the xxx and xxy geometries, respectively. We also took the same image for other possible arrangements of the excitation beams and analyzer polarization, and our observations are summarized in Fig. S8. Because the polarization dependence is always similar for the different cases where one of the three components (*i.e*., pump beam, Stokes beam, and analyzer) is normal to the others, we only studied the SFG signals (I_SFG_) for the xxx and xxy geometries, using the theoretical model for SHG microscopy, namely ${{\rm{{\rm I}}}}_{SFG}\propto A\{{({si}{{n}}^{2}\theta +B{co}{{s}}^{2}\theta )}^{2}+C{si}{{n}}^{2}2\theta \}$, where *A*, *B*, and *C* are fitting constants. The results are summarized in Fig. S7C. The theoretically fitted results on the polarization anisotropy suggest that the myosin substructure in the *C. elegans* muscle is spiraled (α-helix) along the filament axis^27^. On the other hand, to better understand the polarization dependence of the SFG signal, we plotted the unnormalized orientation-dependent signals for 2960 cm^−1^ and calculated the corresponding polarization anisotropy (PA). We found there were negligible levels of differences between the xxx and xxy configurations (Fig. S7D) for both 2860 and 2960 cm^−1^.

**Figure 3:**
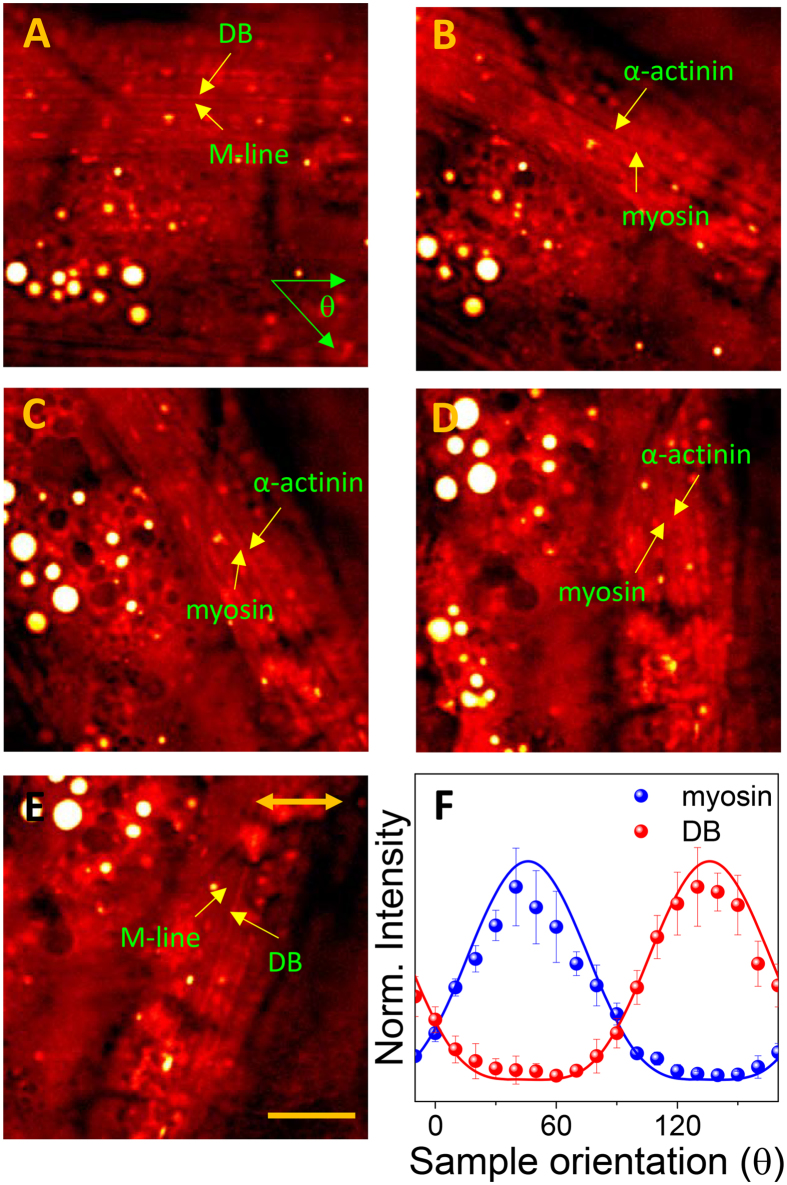
Polarization-dependent appearance of muscle subunits. The spCARS images of body wall muscle near the vulva of WT *C. elegans* for the 2860 cm^−1^ vibration mode, when the angle of the sample with respect to the excitation beam θ was (**A**) 0°, (**B**) 30°, (**C**) 60°, (**D)** 90°, and (**E**) 120°. The scale bar represents 20 μm; Double-sided arrow indicates the direction of the polarization of two incident beams. (F) Polarization of the spCARS myosin (blue) and Z-line (red) signals as a function of the sample orientation.

The polarization dependence of the spCARS signals was studied when the polarization of the analyzer was parallel to the pump and Stokes beams (which are parallel to each other) because the results when the polarization of the analyzer was perpendicular to the beams were not significantly different, except for a slight trend in the absolute spCARS intensity. Figures 3A through 3E show spCARS images of the muscle structures near the vulva, for different values (from 0° to 120°) of the angle θ between the sample and the excitation beam. We can clearly trace the appearance in, and disappearance from, the spCARS images of the DB and myosin with the rotation of the sample (yellow arrows) with a difference of 90°, while the granule-shaped α-actinin (which is at the same position as the DB) is always visible. However, this kind of polarization-dependent spCARS image variation was not observed for every sample, presumably due to sample-to-sample variation and a weak CARS-generating geometry of the muscle fibers. The spCARS contrasts produced by the myosin and the DB were calibrated with respect to the background signal (which was independently acquired from the glass) and normalized in Fig. 3F. The error bars were created from the three data-acquisition locations indicated in Fig. S9, chosen according to the intra-animal location variability in one animal. As shown in Fig. 3F, the polarization dependence of the spCARS signal (for both the myosin and the DB) could be modeled through a cos^6^θ term, which is typically observed for χ^(3)^ nonlinear optical signals. As a consequence, the spCARS signals from the myosin and the DB peaked when each of the structures formed an angle of 45° with the direction of the pump/Stokes beam polarization; thus, the polarization-insensitive α-actinins stand out against the DB especially when the orientation of the DB fiber is close to 0° or 90°.

**Figure 4:**
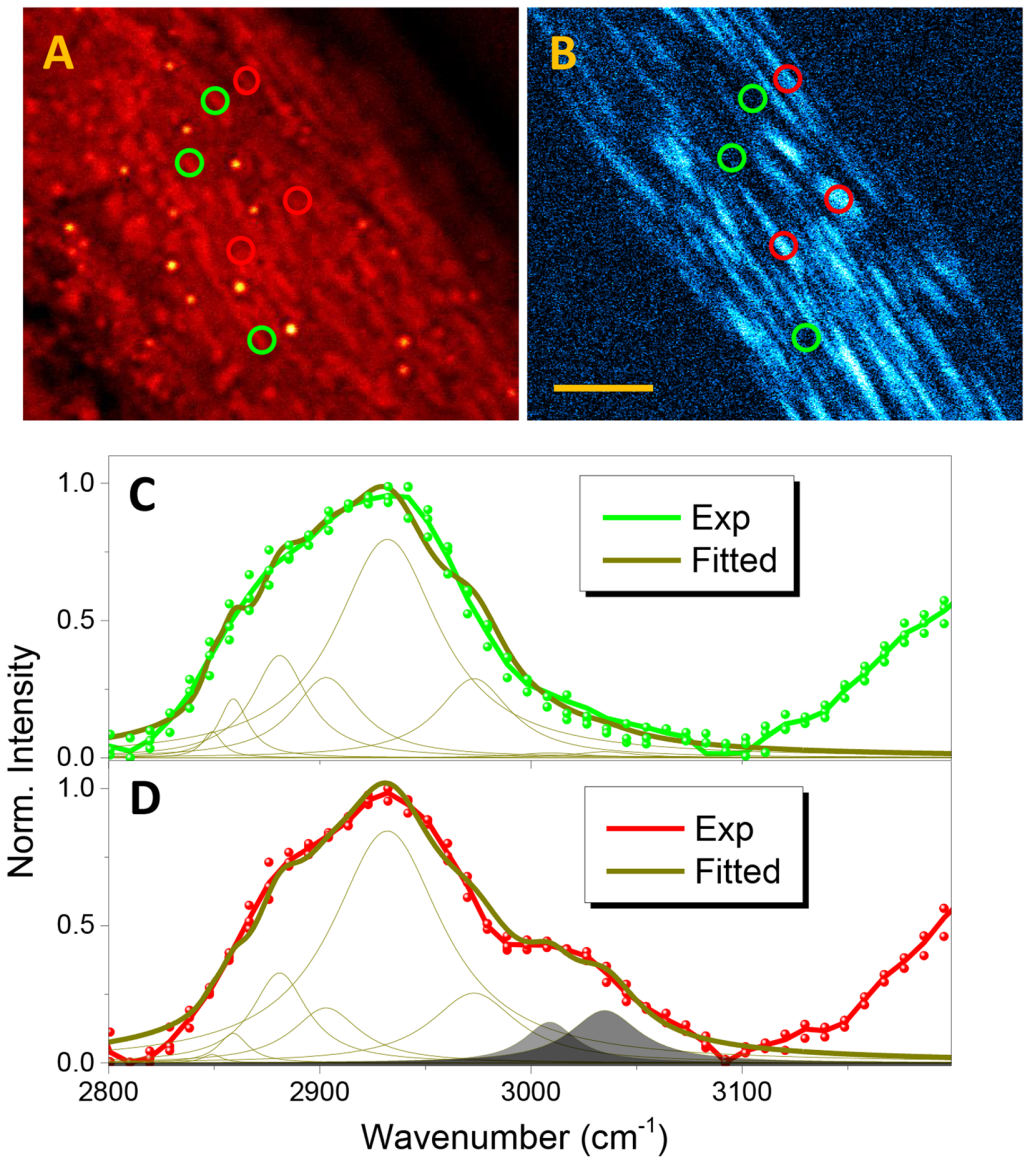
Morphological and chemical changes associated with mutagenesis in the muscle. The (**a**) spCARS and (**b**) SFG images of the body wall muscle of *unc-89* mutant C. elegans, for the 2860 cm^-1^ vibration mode. The scale bar represents 20 μm. (**c**) and (**d**) show the spCARS spectra taken from the green (c) and red (d) circles in (a) and (b).

After numerous imaging tests on several muscle-defective nematodes such as *sqt-3*, *unc-112*, and *dim-1* mutants, we chose *unc-89* (e1460) mutant animals for comparison with the WT strain; *unc-89* encodes a component of the M-line and a key protein for organizing the myosin filament structure of muscles^28^ in *C. elegans*. First, we analyzed the body wall muscle (which experiences the most drastic SFG morphological variation) from an arbitrary region near the oocyte system, as shown in Fig. 4A (spCARS) and 4B (SFG). Overall, we found that spCARS microscopy cannot yield morphological views of straight A-band myosin structures as in WT animals, an effect which also appears for SFG microscopy in the form of frequently interrupted fibrils. To chemically relate the observed spCARS contrasts with the morphological differences, we grouped the target locations according to their SFG brightness into bright (green circles) and dark (red circles) regions. Thus, with the help of SFG contrasts, Fig. 4C,D can be interpreted as the spCARS spectra from myosin-rich and DB-rich structures, and both show a decent increase in the thickness of the congested C–H stretching region (2850–2950 cm^−1^). In summary, the multiple peak deconvolution (Table S2) according to the individual Raman modes shows not only a clear increase in the 2881 cm^−1^ mode but also a dominance in terms of size of the 2932 cm^−1^ mode; a noticeable increase accompanies the 3009 cm^−1^ and 3035 cm^−1^ (one or two trans H-C=C stretches) vibrational modes in the case of the myosin-rich region. The myosin of *C. elegans* is known to exhibit a core-shell type assembly^29^, which should be structurally vulnerable in the absence of a connector, i.e., the M-line, in the case of *unc-89* mutant nematodes. Therefore, the developed structural instability can create an extra element for modulation in the secondary structure of the aliphatic myosin protein, leading to an additional enhancement of the spCARS intensity at the right shoulder of the C-H stretching peak^30^.

**Figure 5:**
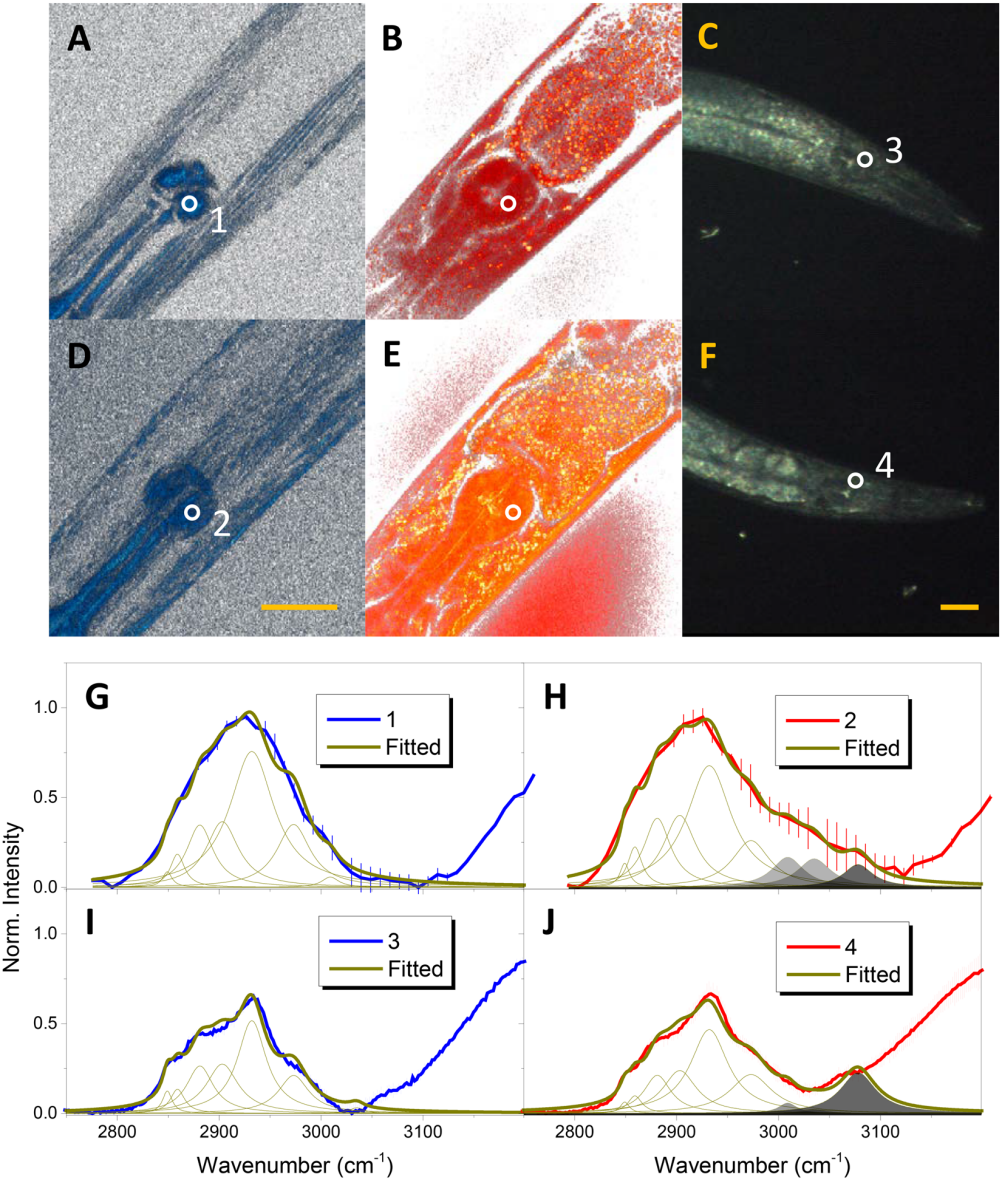
Effects of the variation of secondary structure on spCARS. Merge of the 3D stack of (**A**) SFG and (**B**) spCARS images of the TB of WT *C. elegans*, for the 2860 cm^−1^ vibration mode. **(C)** Dark-field image of an arbitrary WT worm. **(D,E,F)** are the analogues of (**A,B,C**), respectively, for *unc-89 C. elegans*. The scale bars represent 30 μm. **(G)** and **(H)** show the spCARS spectra of (**G**) WT and (**H**) *unc-89 C. elegans* for the positions 1 and 2 in (**A**) and (**D**). Similarly, (**I**) and (**J**) show the spontaneous Raman spectra of (**I**) WT and (**J**) *unc-89 C. elegans* for the positions 3 and 4 in (**C**) and (**F**).

We also employed spontaneous Raman spectroscopy (spRS) to critically assess the credibility of the vibrational modes of the acquired spCARS spectra. Figure 5A,B,D and E show the difference between the SFG and spCARS contrasts, illustrating that the terminal bulbs (TBs) of nematodes contain a limited amount of lipids within the level of spatial resolution of spRS (~4 μm: lateral, >10 μm axial). In this sense, we selected the TBs as the target positions to compare the spCARS and spRS spectra for the chemical composition analysis, and the spRS spectra for the WT and *unc-89* mutant animals were taken at the circled areas shown in the dark field images (Fig. 5C,F). We performed spCARS (1, 2) and spRS (3, 4) spectroscopy and the results are presented in Fig. 5G to 5I. Basically, in the WT case, the spCARS spectrum (Fig. 5G) showed a clearly larger amount of 2881 cm^−1^ modes in the TB (peak area of 17.4, in arbitrary units) than in the body wall muscle (3.0). A similar result was found in the case of spRS spectrum, with an area of 13.3 for the TB (Fig. 5I, Table S3). As previously discussed for the lipid droplet, we can assume that the TB likely has ordered C-H structures, which can promote the Fermi resonance between stretching and bending motions. In the case of the spCARS spectrum for the TB of the *unc-89* mutant nematode (Fig. 5H), the Raman linewidths for the different vibration modes were quite similar to those for the WT worm except for the 3009 and 3035 cm^−1^ modes (which here were found to be 10.1 and 9.8, while for the WT strain were fitted to 3.1 and 2.1, respectively). A similar tendency of the individual Raman mode contribution is observed for the TB of WT and *unc-89* animals in Fig. 5I,J, even up to the 3009 and 3035 cm^−1^ modes in the case of spRS spectra. Intriguingly, we had to use an additional vibration mode at ~3078 cm^−1^, which corresponds to an amide B bond, with considerable amplitudes (7.1 and 14.8 for spCARS and spRS, respectively) to better fit the experimental data of *unc-89*. Concerning the appearance of the amide B band in the *unc-8*9 TB, it has been reported in the literature^31^ that the Fermi resonance can play a role in enhancing the interplay between amide A (~3326 cm^−1^) and the overtone of the amide II band (~1549 cm^−1^)^32, 33^. Thus, we also traced the fingerprint region of the spRS spectra for WT and *unc-89* worms, revealing a distinctive increase in the amide II band in the case of *unc-89* (Fig. S10). Further, Fig. S10 provides additional information that suggests the appearance of tryptophan (~750 cm^−1^), the unfolding of an α-helix structure^34^ (amide I), and the augmentation of the C-N stretching modes in the TB of the *unc-89* mutant^35^. The two latter phenomena can be explained as a possible promoted denaturation and an extra degree of freedom for the movement of the myosin fiber in the absence of the M-line, which otherwise tightly holds myosin filaments. The Raman intensity of tryptophan residues is also known to be an indicator for the variation in the tertiary protein structure, which increases as the structure gets more concealed; thus, some changes in the tertiary structure prevent the tryptophan residue in *unc-89* mutant animals from being exposed.

**Figure 6:**
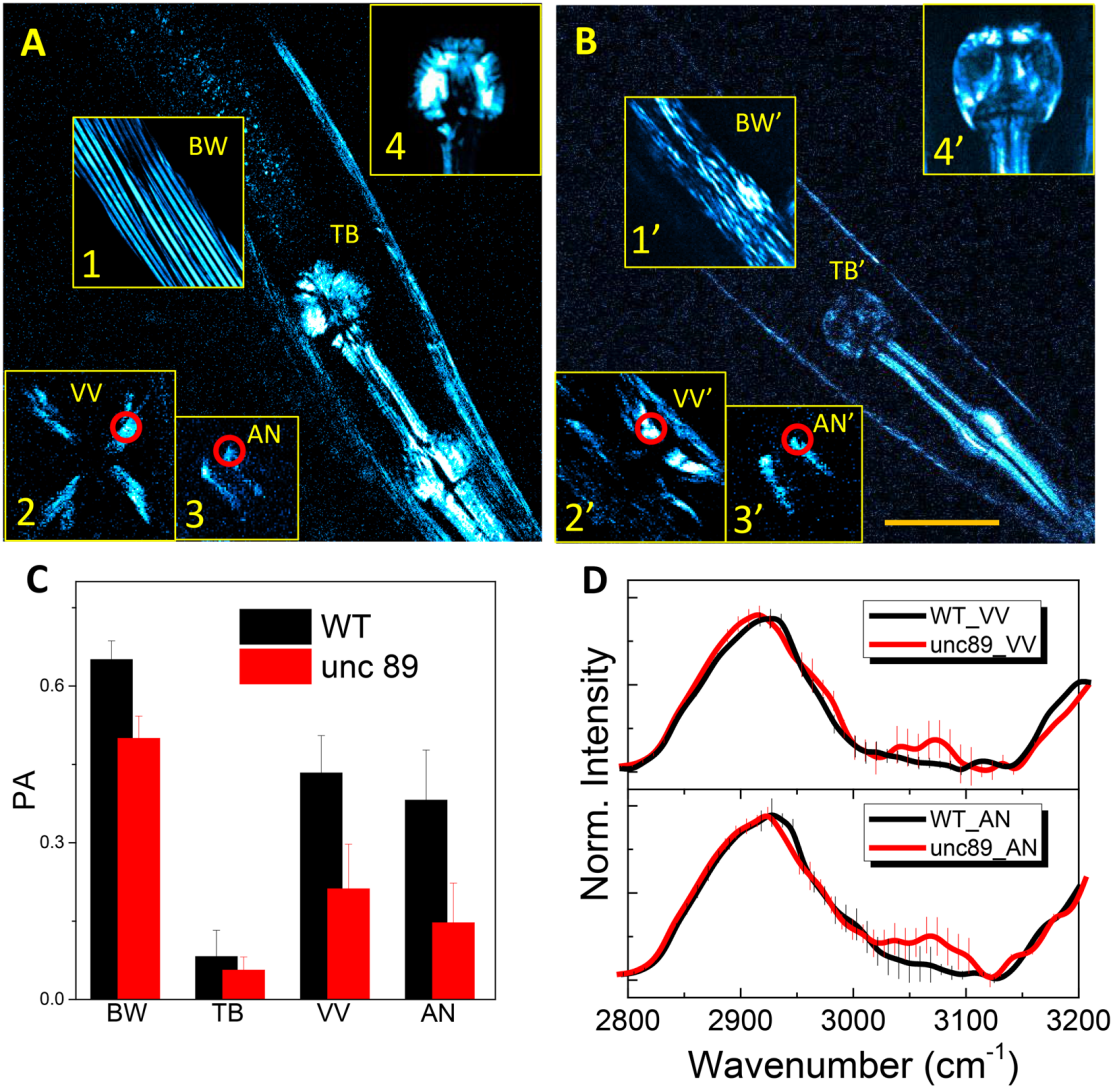
Morphochemical changes associated with mutagenesis in various muscle organs. SFG images of pharyngeal muscles for (**A**) WT and (**B**) *unc-89* mutant animals. The scale bar represents 50 μm. The inset images correspond to the body wall muscle (1), vulva (2), anus (3), and terminal bulb (4) (a prime sign indicates that the corresponding organs are from the *unc-89* strain). (**C**) Comparison of the PA (polarization anisotropy) of various muscles between the *unc-89* mutant and WT strains. (**D**) spCARS spectra for the red circles in the insets of (**A**) and (**B**)(VV: vulva, AN: anus).

Since SFG microscopy enables quick and straightforward 3D imaging on the *in vivo* muscle regardless of the sample geometry, it was adopted to investigate the morphological changes in the muscle of the *unc-89* mutant animals compared to the WT worms. To do so, we obtained an overall view of *C. elegans* by patching the panoramic SFG images seen in Fig. S11A,B (reformatted using the illumination-assisted 3D volume viewer in the Image J software). We could project the general tendency of low SFG signals over the entire body of *the unc-89* mutant animals, as well as the WT, regarding the pharyngeal, body wall, and vulval muscle, and even intestinal granules spread over the digestive organs. More specifically, the morphological shape of the terminal bulb of the *unc-89* mutant animals could be largely classified as selectively less distinct (even less than the 1st bulb) and there was no “cilia-shaped” structure at the boundary (Fig. 6A,B). Another prominent morphological phenotype among the *unc-89* mutant animals was seen in the body wall muscle, which was totally discontinued, distorted, and even aggregated as scattered spherical structures showing beam-polarization independent SFG responses (inset 1’ in Fig. 6B). In addition, the vulval muscle of the *unc-89* mutant animals was asymmetrically disoriented, with higher chances of reversing the sample diversity, as shown in Fig. S11C,D. It is also intriguing that the SFG contrasts under xxy geometrical conditions almost totally obscured anal (3) and pharyngeal (4) muscles regardless of whether the WT or *unc-89* strain was used. The polarization anisotropy (PA) values for the organs analyzed allowed for the observation of some interesting features; in particular, there was a general tendency for some features of the *unc-89* strain to be altered, including a dramatic disappearance (<0.1) of the terminal bulb (Fig. 6C). The error bars here were from 7 animals due to the inter-animal variability. This phenomenon is presumably due to the round structural morphology. Additionally, the PA decrease of the *unc-89* strain compared with the WT was more severe at the vulva and anus. The spCARS spectra from the target area were also traced as shown in Fig. 6D, suggesting a spectral raising at the tail of the C-H mode around 3000–3100 cm^−1^, attributed to amide B band both for the muscle of the anus and vulva regions of the *unc-89* mutant animals. A localized increase in the case of the vulval muscle spectra is slightly less dominant than that for the anal muscle. The appearance of amide B band can be explained with the enhanced Fermi resonance while the slight difference in the width of the congested C-H vibration modes can be also potentially linked to the peak broadening of the unfolded polymer chains^36^ as well as enhanced tryptophan concealing due to the modification of the tertiary structure as mentioned above. Still, we believe that our phase-sensitive spectral adjustment technique of modified Kramers–Krӧnig transform method could more critically resolve the discrepancy of the spectra after tracing the averages of 7 target images with the possibility of a sample-to-sample variation being expressed in the newly traced spectra from different animals.

**Figure 7:**
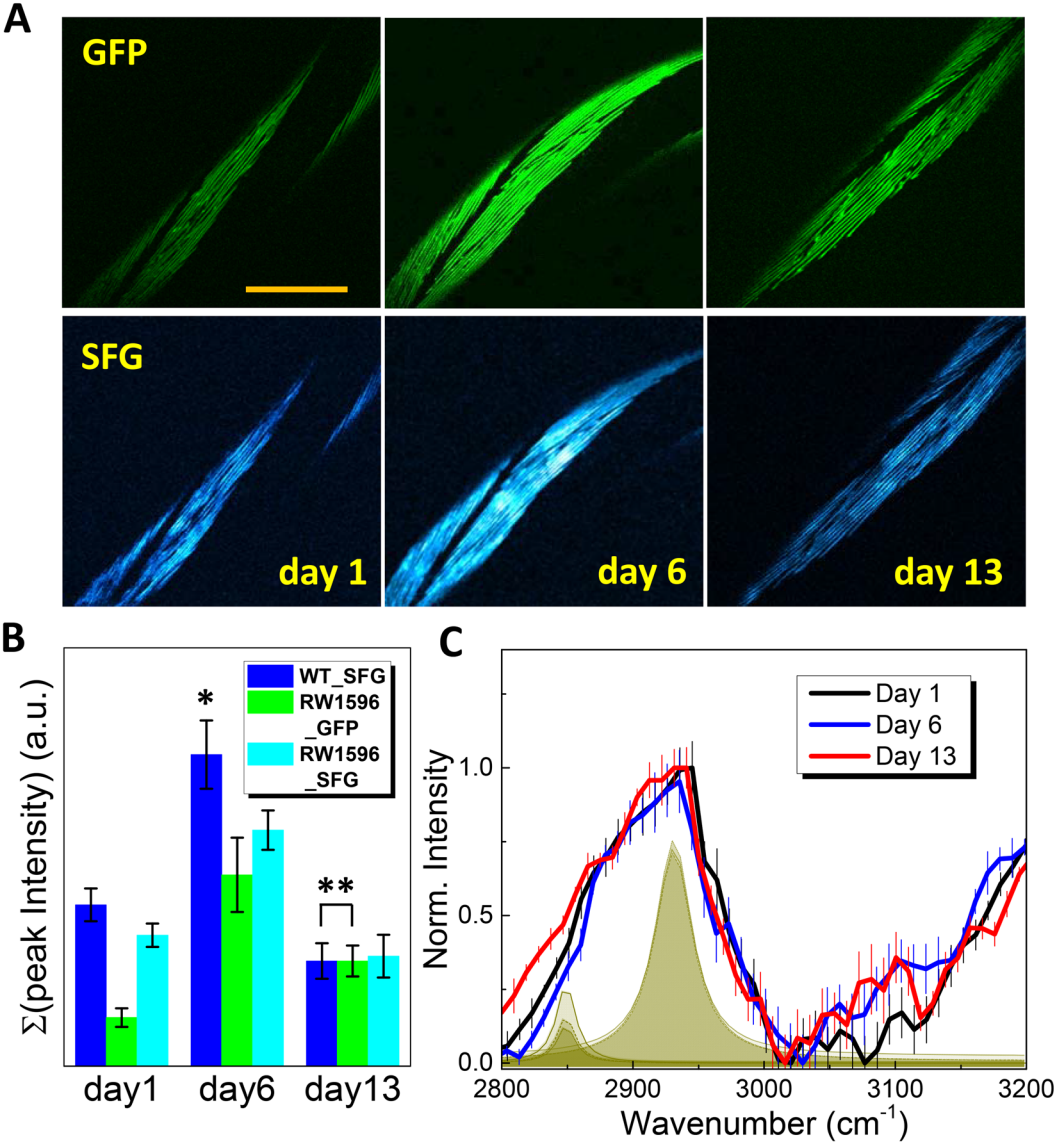
Spectrally focused CARS/SFG microspectroscopy as a technique to study muscle aging. (**A**) Two-photon excited luminescence of green fluorescent protein (GFP, upper panel) and SFG (lower panel) images of the body wall muscle in variously aged (1, 6, and 13 day-adulthood) RW1596 mutant animals. The scale bar represents 50 μm. (**B**) Comparison of the summed peak intensity for the SFG and GFP images of WT and RW1596 mutant animals. The value of * was obtained from 7-day old WT worms. ** Two values are factored to be identical while the ratios among three intensity values from WT animals are retained. (**C**) Normalized spCARS intensity for 1-, 6-, and 13-day-old RW1596 mutant animals.

To confirm the validity of SFG microscopy assisted by spCARS spectroscopy to study muscle aging in *C. elegans*, we need to achieve a positive rationale regarding the specific location of the SFG site within the *C. elegans* muscle. To this end, we investigated the correlative nature of “unidentified” SFG images with the commonly accepted fluorescence microscopy of mitochondria, nucleus, actin and myosin, either expressing green fluorescent protein (GFP) or labeled with dye molecules. First, the co-localization between SFG and two photon excited fluorescence (TPEF) signals from the GFP expressed in the mitochondria and nucleus of strain PD4251 was compared (Fig. S12A). We were able to confirm that the appearance of the SFG/GFP contrast was closely localized to the body wall muscle. Another GFP-conjugated animal, RW1596, allowed GFP to be expressed at the myosin filament level in the body wall muscle. The z-directional sectioning of *C. elegans* passing through the terminal bulb and providing a plane view revealed that the degree of co-localization was higher in the PD4251 strain (Fig. S12B,C). Furthermore, the SFG/GFP line profiles (Fig. S12D) along the dashed lines in Fig. S1D quantitatively suggest that the signal correlation between SFG and GFP is strong (R2 = 0.77). Finally, the position of the actin site was also compared with the SFG-bright site shown in Fig. S13. The labeling of actin was performed as previously described^37^ and we could conclude that the position of the green (by actin) was discriminated from that of the red (by SFG). From these data, we found that the myosin would be the major structural source for the generated SFG signals as in SHG^27^. Because the RW1596 strain contains the myo-3::GFP, which is known to decrease with aging^38^, we performed a direct imaging comparison between GFP and SFG, as shown in Fig. 7A. A quantitative comparison obtained by summating the intensity histograms (Fig. S14) over 10 blindly taken GFP/SFG images (animal-to-animal variability) from similar-sized RW1596 strains (which are all RW1596 isolates) and SFG from WT (Fig. 7B) strongly suggests a positive correlation between the GFP and SFG signals, leading to the possibility of using SFG microscopy as a tool for muscle aging studies in *C. elegans*. Figure 7C shows the phase-retrieved spCARS spectra for three aged RW1596 stains. Compared with day-1 adulthood, which shows a similar spectral tendency to the WT, we can clearly observe a distinct rise over a short range of 2800–2900 cm^−1^ vibration modes at day13 adulthood by a factor of ~2 with respect to the deconvoluted amplitude ratio at the center of 2849 cm^−1^ (5.1 for 1 day; 4.5 for 6 days; 10.4 for 13 days), while those from day-6 adulthood were retained almost the same. This observation did not exactly match the tendency for the variation in the amount of “myosin” seen in Fig. 7B with the values being reversed between the day-1 and day-13 samples (although it matches the day 6 sample). Only a limited number of studies allow for possible explanation of our observation by the augmented fat infiltration to the muscular systems with aging^39^. Nevertheless, we believe that the alteration in the spCARS spectra, apparently from a combined contribution of the volume and chemical composition of myosin, was qualitatively correlated with aging.

## Conclusion

In summary, we performed *in vivo* spCARS imaging of *C. elegans* combined with SFG microscopy by mixing two spectrally focused femtosecond pulsed lasers. Both muscle components such as myosin or the DB and non-muscle-related lipid droplets could be neatly visualized using spCARS microspectroscopy, and also (in the presence of the solid myosin position marker) by SFG microscopy, for any sample orientation. The C-H vibration and mixed vibration modes in the 3000–3200 cm^−1^ range were well resolved by eliminating the terms that are not vibrationally resonant from the spCARS spectra through a modified KK transformation method. Intriguingly, the polarization dependence of the spCARS microscopy showed that the myosin and the DB of the muscle helical fibers were approximately perpendicular; also, round-shaped α-actinin structures were resolved from the DB when the beams polarization formed an angle of 45° with the DB fibril direction. We obtained evidence for changes in the chemical composition for the 2800–3100 cm^−1^ vibration modes using the myosin-defective mutant (*unc-89*), although such changes were as obvious as those affecting the morphological shape for the body wall and TB. We also determined that spCARS/SFG microscopy is a useful tool to quantitatively estimate the amount of myosin, which is an aging-sensitive biomarker possibly associated with the existence of aging-related fat, when combined with the chemical information for the ~2800–2900 cm^−1^ vibration modes. We believe that our results, obtained by visualizing the muscle sarcomere through SFG (myosin) and spCARS (myosin, M-line, DB, α-actinin, and adjacent organelles), combined with chemical information, are technically very novel and conceptually sound.

## Electronic supplementary material

Supplementary information
