# Supplementary material for: Coherent Raman Imaging of Live Muscle Sarcomeres Assisted by SFG Microscopy
Source: Sci Rep. 2017 Aug 23;7:9211. doi: 10.1038/s41598-017-09571-w (PMC5569110; doi:10.1038/s41598-017-09571-w)
Supplement: Supplementary file 1 — Supplementary information [file 41598_2017_9571_MOESM1_ESM.pdf]

Supporting Information for

Coherent Raman Imaging of Live Muscle Sarcomeres Assisted by SFG Microscopy

Hyunmin Kim[1,*,†], Do-Young Kim[2,†], Kyung-Il Joo[3], Jung-Hye Kim[1], Soon Moon Jeong[1], Eun Seong Lee[4], Jeong-Hoon Hahm[5], Kyuhyung Kim[2], Dae Won Moon[6,*]

[1]Companion Diagnostics & Medical Technology Research Group, DGIST, Daegu 42988, South Korea.
[2]Department of Brain and Cognitive Science, DGIST, Daegu, 42988, South Korea.
[3]Department of Electronics Engineering, Kyungpook National University, Daegu 41566, Republic of Korea.
[4]Center for Nanometrology, Korea Research Institute of Standards and Science, 267 Gajeong-ro, Yuseong-gu, Daejeon, 34113, South Korea.
[5]Center for Plant Aging Research, Institute for Basic Science, Daegu, 42988, Republic of Korea.
[6]Department of New Biology, DGIST, Daegu, 42988, South Korea.

†These authors contributed equally to this work.
*Corresponding authors: hyunmin.kim@dgist.ac.kr and dwmoon@dgist.ac.kr

Construction of the spectrally focused CARS (spCARS) microscope

Figure S1a shows a schematic of the temporal overlap of two incident photons (P and S represent the pump and Stokes beams, respectively) as a function of the interpulse delay. The inset energy diagram conceptually represents the two relevant nonlinear optical wave-mixing processes (i.e., spCARS and SFG). We can see that, for spCARS, the energy difference between the two pulses is tuned to the molecular vibration of interest. The inspiration for the current setup, which combines spCARS with SFG microscopy[1] (as illustrated in Figure S1b), was provided by a spectral-focusing hyperspectral coherent Raman scattering (CRS) microscope system[2] in which the target vibrational mode is studied by introducing a time delay between the pulses, instead of tuning their wavelength difference. In our system, one of the two ultrafast femtosecond (80 MHz) pulse trains from a commercial mode-locked erbium-doped fiber laser (InSight DeepSee Dual, Spectra-Physics) served as a variable wavelength pump beam (120 fs, 680–1300 nm) and the other as the Stokes beam (220 fs, 1041 nm). The full width at half maximum (FWHM) of the pump and Stokes beams was measured to be ~7 and 8 nm, respectively. To minimize sample damage, the pulse power at the sample was kept at ~8 mW for the pump beam and ~30 mW for the Stokes beam using a half-wave plate (ACWP-700-1000 for the pump beam and PTOL-ACWP-1000-1600 for the Stokes beam, CVI) and Glan–Thompson polarizers (PTOL-10.0-670-1064, CVI). The two beams were collinearly aligned with a dichroic mirror (DMSP1000R, Thorlabs), and their temporal overlap was realized using a motorized translation stage (SGSP46-500, Sigma-Koki). Then, the two overlapped beams were simultaneously stretched by a 24-cm-long SF57 glass square-column (composed of two 12-cm-long glasses), so that the FWHM of the net time-dependent spCARS beam profile reached ~1.0 ps due to the pulse chirping. The inherent chirping of pump pulse caused by the various optical components in this setup was measured to be ~0.08 ps.

In order to create a real-time 3D image, the two overlapped beams were sent to a galvanometric scanning system (FluoView 1000, Olympus) equipped with an inverted microscope (IX83, Olympus) and then focused onto the sample through an oil-immersion objective lens (UPlanFLN, Olympus) with a numerical aperture (NA) of 1.35. The spCARS/SFG signals generated from the *C. elegans* muscle were collected by a photomultiplier tube (R3896 PMT, Hamamatsu) after passing through a condenser (NA = 0.55), a Glan-Thompson analyzer (PTOL-10.0-425-675, CVI), and bandpass filters (FF02-460/80 for SFG and FF01-650/60 for spCARS, Semrock). The backscattered signals were also collected in the epi direction using a PMT detector for two-photon-excited fluorescence (TPEF) imaging. Then, they were separated using a dichroic mirror (RDM690, Olympus) and signalized by the combined use of a monochromator (Acton SP2300, Princeton Instruments) and a CCD camera (PIXIS 100B, Princeton Instruments) for broad wavelength scanning, including spCARS/SFG. For the wavenumber calibration, the spCARS spectra of dimethylsulfoxide (DMSO) was rigorously examined by moving the relative temporal position (10 μm) of the pump beam with regards to the Stokes beam. By using a forward PMT detector, we profiled the spCARS spectrum based on serial images of a DMSO solution (red balls) and compared it with spontaneous Raman spectrum (black line) as suggested in Fig. S2a. The chirp was calibrated to ~0.53 $cm^{-1}/\mu m$, which matches the value found in the literature (~0.55 $cm^{-1}/\mu m$) for a 25.4-cm-long SF57 glass rod.[3] The spectral positions of the displayed spCARS thumbnail spCARS images of DMSO were adjusted using the reference peaks located at 2913 and 2996 cm^{-1}. These peaks served to calibrate the system for measuring the molecular vibration modes of proteins (2850–3150 cm^{-1}) in the muscle tissue.

Phase-retrieval of spCARS spectra

In Figure S5a, spCARS spectra (solid lines) were fitted over the nonresonant background spectra (dotted lines) from the glass to more selectively retrieve vibrationally sensitive terms, when the pump wavelength was tuned to 2894, 2956, 3020, and 3084 cm^{-1}. Figure S5b was obtained by taking the ratios of the resonant and nonresonant signals of Figure S5a. The more significant contributions from the background signals in Figure S5a occurred at the profile center when the pump beams were tuned to 2956 and 3020 cm^{-1}, possibly because for they are the wavenumbers the objects were vibrationally less sensitive. The wavenumber range (2800 cm^{-1}–3000 cm^{-1}) and spCARS intensity profiles displayed in Figure S5b agree quite well with other results in the literature[4,5] without considering phase-correction procedures. Figure S5c shows the spCARS spectra retrieved from the body wall muscle of *C. elegans* after factoring out the influence of the background signals using the modified Kramers–Krönig (KK) transformation method[6]; here the pump wavelength was tuned to the same vibration modes as in Figure S5a.

Figure S6 shows the details of the image-based phase-retrieval process used to extract the spCARS signals in Figure S5, for one of the wavelengths (the 792 nm pump excitation). The final form of the spectra was obtained by patching the spectrum from 2700 cm^{-1} to 3000 cm^{-1} obtained with the 802 nm pump (2860 cm^{-1} center) and that from 2900 cm^{-1} to 3200 cm^{-1} obtained with the 792 nm pump (3020 cm^{-1} center). We can clearly see a high signal-to-noise level ($S/N_{spCARS} > 100$) in the peaks resolved near the C-H (2850–2950 cm^{-1})[7] and N-H (>3000 cm^{-1})[8] vibration regions, where the FWHM of the spCARS spectrum in the 2930 cm^{-1} mode is as broad as

~130 cm^{-1}. The spectra in Figure S5c were drawn from the average values in Figure S5a and showed a significant sample-to-sample variation in the intensity and wavenumber for each resolved vibrational mode (which will be formally expressed through error bars in the following spectral figures). This variation was ascribed to the species diversity, while there was a consistent observation of the tails of the C-H vibration modes and/or overtones of amide II (or amide B) bands at 3000-3100 cm^{-1}.[9]

Concerning the mismatch between turning points toward the high-frequency region (above 3000 cm^{-1}) in Figures S5b and S5c, it is possible that an error factor was introduced through the positioning of the attaching curve during the phase-detrending algorithm, as the plateau in the spCARS intensity seems to indicate in Figure S6e. Nevertheless, it should be noted that the vibrational modes at 3100–3200 cm^{-1} can also be assumed to be a combination of the tails of the amide A and O-H stretching associated with water content. The spectral dependence of spCARS was also evaluated by profiling sequentially collected CARS signals as a function of the pump wavelength. This method showed a significantly reduced chemical sensitivity (with a noticeable ambiguity in the position of the resonance) compared to spCARS, presumably due to the power instability associated with the variation of the wavelength and also to the sample regaining consciousness as a result of the long data-acquisition time.

Spontaneous Raman spectroscopy

To confirm the vibrational modes obtained through the spCARS method, we also performed spontaneous Raman spectroscopy on the terminal bulb (TB) region of the WT and *unc-89* pharyngeal muscle. All Raman experiments were carried out using a commercial Raman spectrometer (Nicolet, Almeca XR) equipped with an 50× objective lens (Olympus, Mplan N) with NA = 0.75, at an illumination wavelength of 532 nm and a power of ~2 mW for 100 s.

Supporting figures

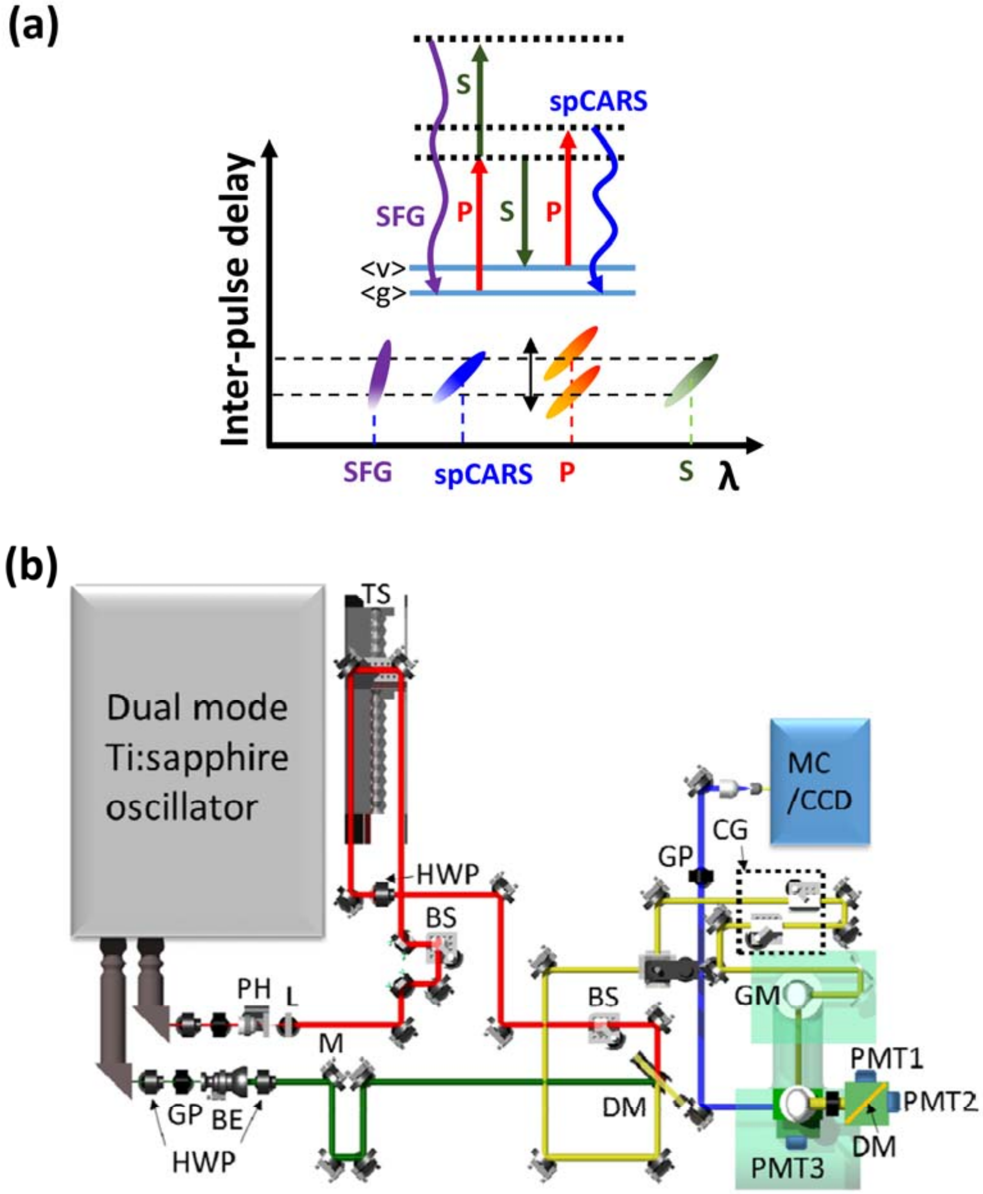

Figure S1. Optical setup. (a) Schematic of the time-wavelength correlation and energy diagram for the spCARS process, which is driven by the temporal overlap of two chirped femtosecond pulses and is inherently accompanied by nonlinear optical SFG. (Here <g> and <v> are the vibrational ground and excited states, while P and S are the pump and Stokes beams.) **(b)** Schematic of the optical setup for the dual-mode SFG/spCARS microscope used in this work. (HWP: half-wave plate; GP: Glan-Thompson polarizer; BE: beam expander; PH: pin hole; L: lens; M: silver mirror; BS: beam splitter; TS: mechanical translational stage; DM: dichroic mirror; CG: chirping glass; GM: galvanometric mirror; PMT: photomultiplier tube; MC/CCD: monochromator/charge coupled device.)

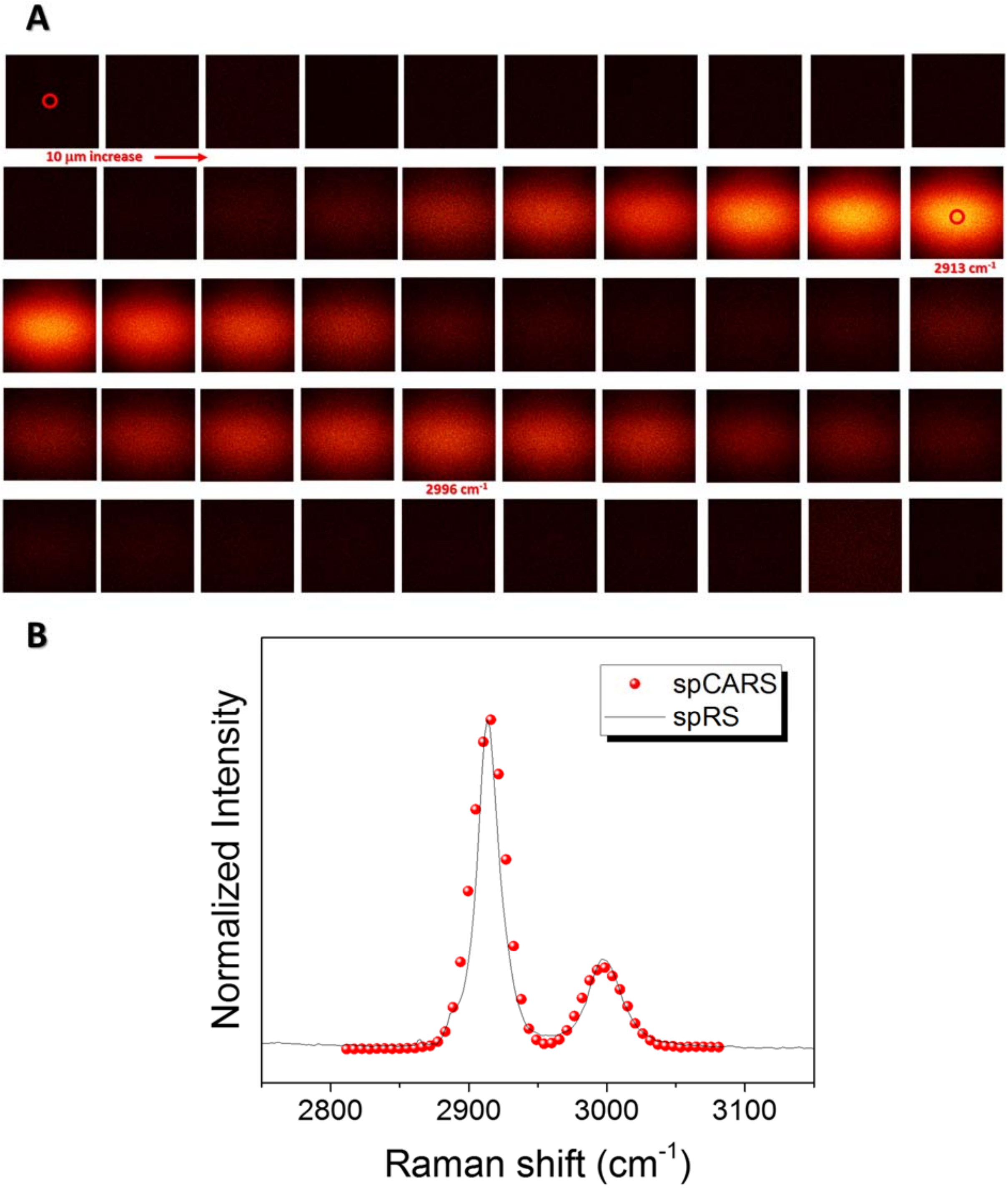

Figure S2. Wavenumber calibration of spCARS spectra using DMSO. (a) Spectrally focused CARS images (thumbnail images) of the DMSO solution as a function of the interpulse delay (or, equivalently, the wavenumber), for a fixed P beam wavelength (792 nm) and a fixed S beam wavelength (1041 nm). (b) The spCARS profile (red ball) traced along the red circle marked in the first thumbnail image and was compared with spontaneous Raman spectra (black line).

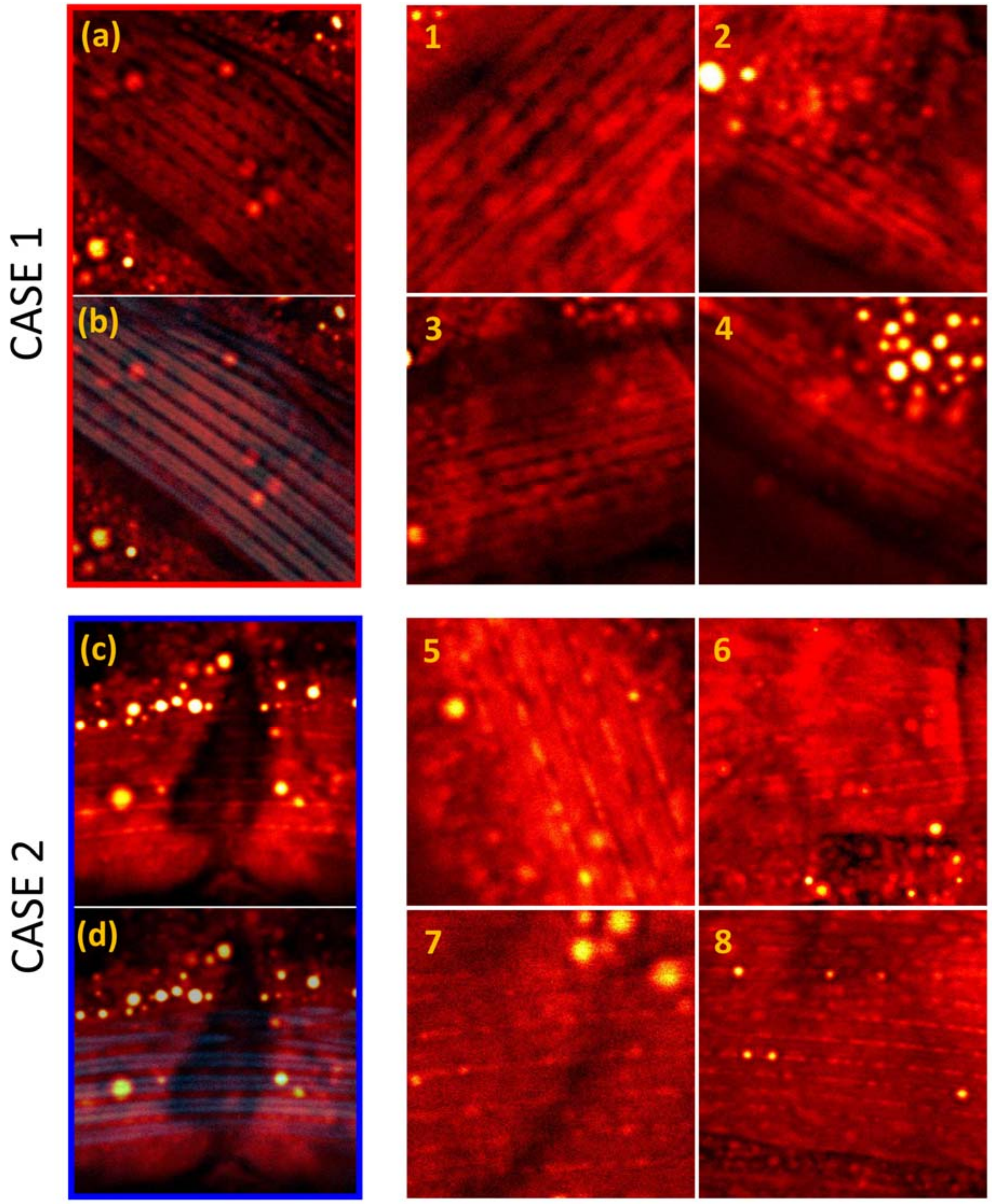

Figure S3. Spectrally focused CARS contrasts from body wall muscle located near the vulva of *C. elegans*. The spCARS contrasts in case 1 were produced from myosin, while those in case 2 were mainly from the DB (and/or M-line and α-actinin). The subunits in the spCARS images (a) and (c) were visually confirmed using the overlay (spCARS + SFG) images (b) and (d), respectively. Images 1 through 4 correspond to case 1, and 5 through 8 to case 2. Scale bars are arbitrary.

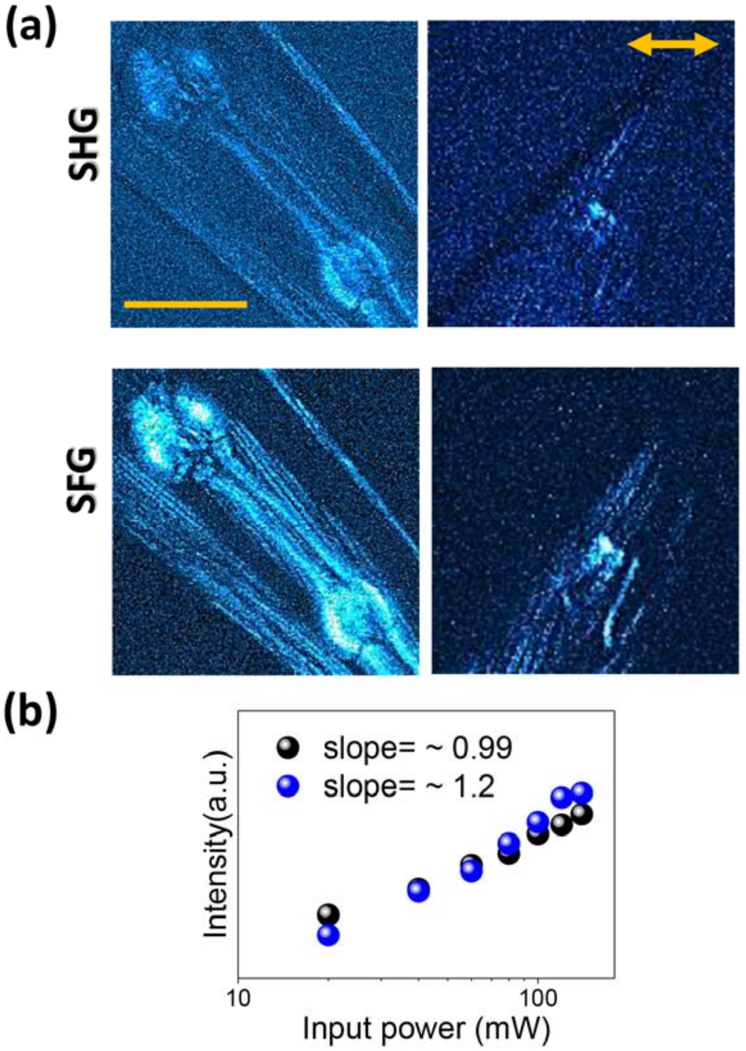

Figure S4. Comparison between SFG and SHG microscopy of *C. elegans*. (a) Images of muscular structures of WT *C. elegans* around the pharynx (left panel) and anus (right panel) using SHG (P: 38 mW) and SFG (P: 8 mW; S: 30 mW; P and S represent the pump and Stokes beams, respectively). The scale bar represents 30 µm. SHG images were taken at a wavelength of ~392 nm using a commercially available filter (FF01-370/50, Semrock). The arrow in (a) indicates the polarization direction of the two beams (P and S) and a polarizer located right before the detector (unless otherwise indicated, the three are parallel). **(b)** Power dependence of SFG intensity for the P (blue) and S (black) beams.

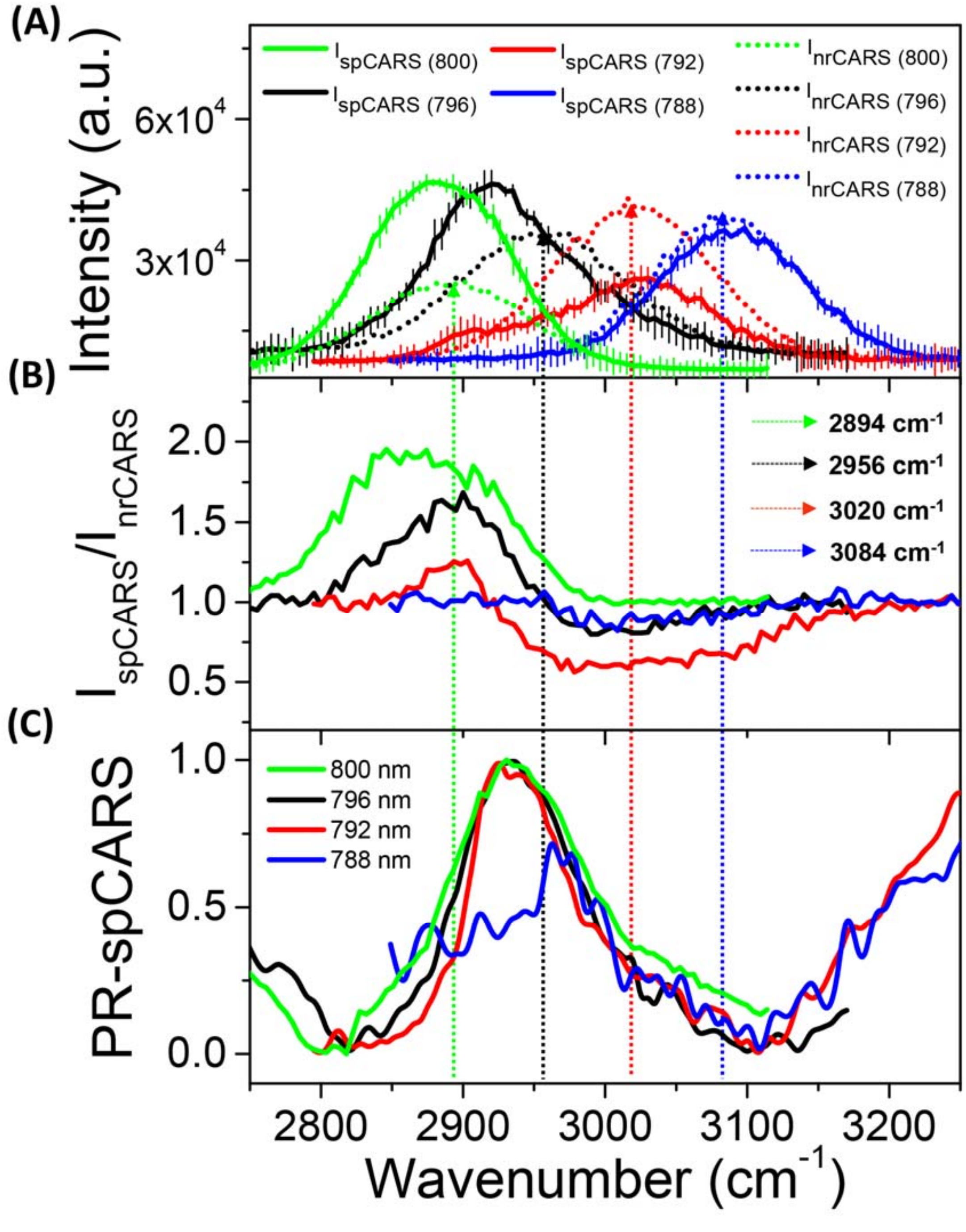

Figure S5. Process for obtaining phase-retrieved spCARS spectra. (a) The spCARS spectra collected when P is 800 nm (2894 cm^{-1}, green), 796 nm (2956 cm^{-1}, black), 792 nm (3020 cm^{-1}, red) and 788 nm (3084 cm^{-1}, blue). The solid lines correspond to myosin structure while the dotted lines are from the glass background (FWHM = 130 cm^{-1}). **(b)** Ratio between the solid and dotted signals in (a). **(c)** Normalized phase-retrieved spCARS signals using the sample and reference signals in (a).

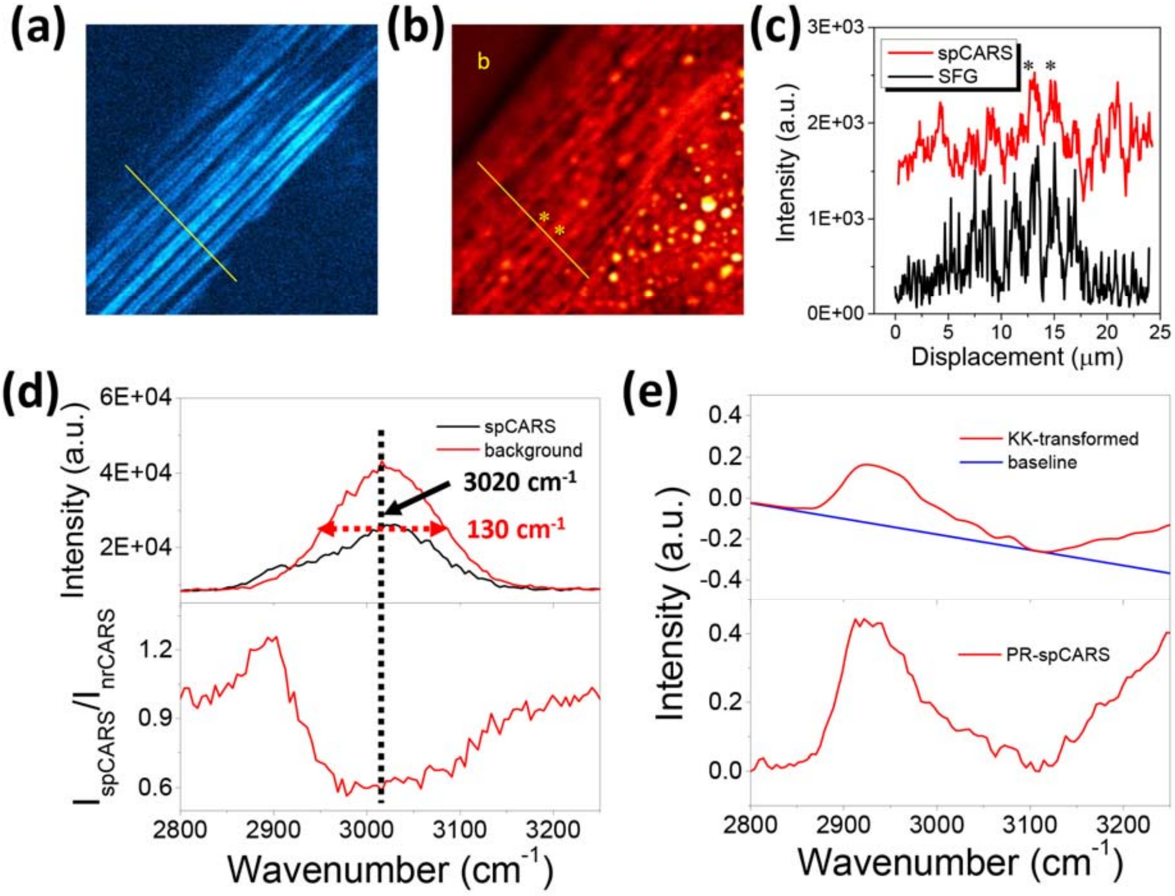

Figure S6. Extraction of resonant spCARS spectra using a phase-retrieval method based on the modified KK transformation. **(a)** SFG, **(b)** spCARS, and **(c)** overlay images of "myosin-detectable" body wall muscle of WT *C. elegans* when P is 792 nm. The scale represents 20 μm. **(d)** spCARS spectra (upper panel) taken from the black (resonant term) and red (non-resonant) star positions in (a) and (b). The ratio between those spectra is shown in the lower panel. **(e)** Raman-like spectrum (upper panel) after taking the KK transformation of (d). The lower panel shows the final form of the phase-retrieved spectrum after detrending (removing the baseline signal).

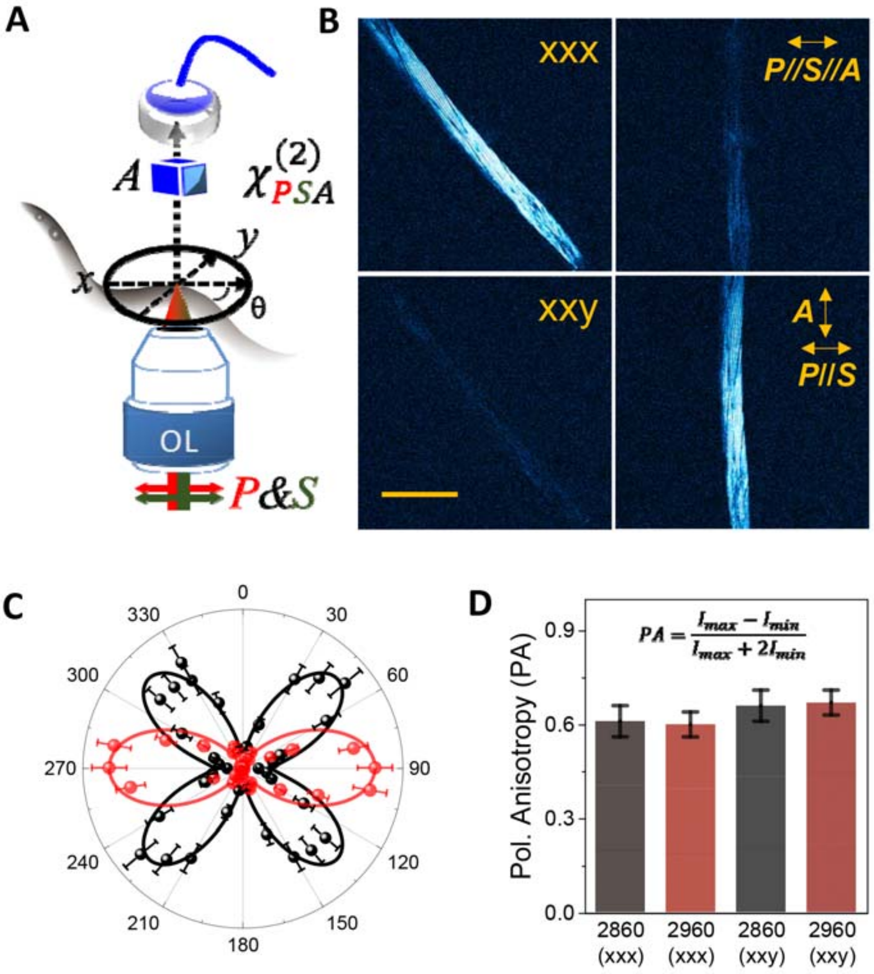

Figure S7. Polarization dependence of the SFG microscopy of *C. elegans*. (a) Sketch of the orientation of the *C. elegans* sample with respect to the polarizations of the incident beams (P and S) and analyzer (A). **(b)** SFG images (dependent on the orientation of the sample) of WT *C. elegans* when the polarization of the analyzer was parallel (xxx, upper panel) and normal (xxy, lower panel) to that of the incident beams. The scale bar represents 50 µm. **(c)** Polarization dependence of the SFG for the xxx (black) and xxy (red) geometries. **(d)** Polarization anisotropy (PA) for the xxx and xxy geometries when P is tuned to 2860 and 2960 cm^{-1}.

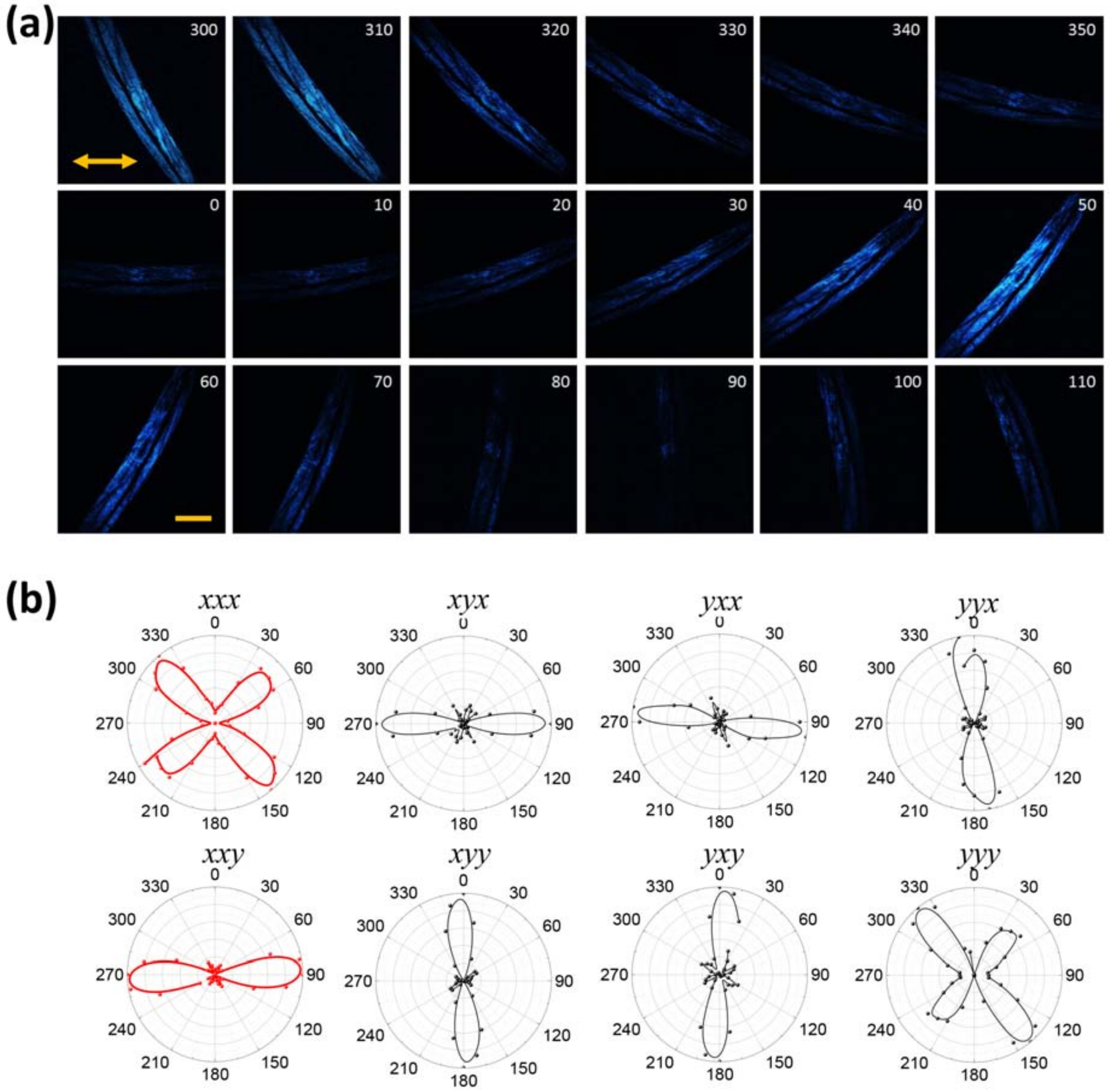

Figure S8. SFG images of *C. elegans* for various polarization arrangements. (a) Series of SFG images of a WT *C. elegans* sample rotated about the scanning center. The scale bar represents 50 μm. **(b)** Normalized polarization-dependent SFG pole figures for different arrangements of the P, S, and analyzer polarizations (xxx, xyx, yxx, …, yyy).

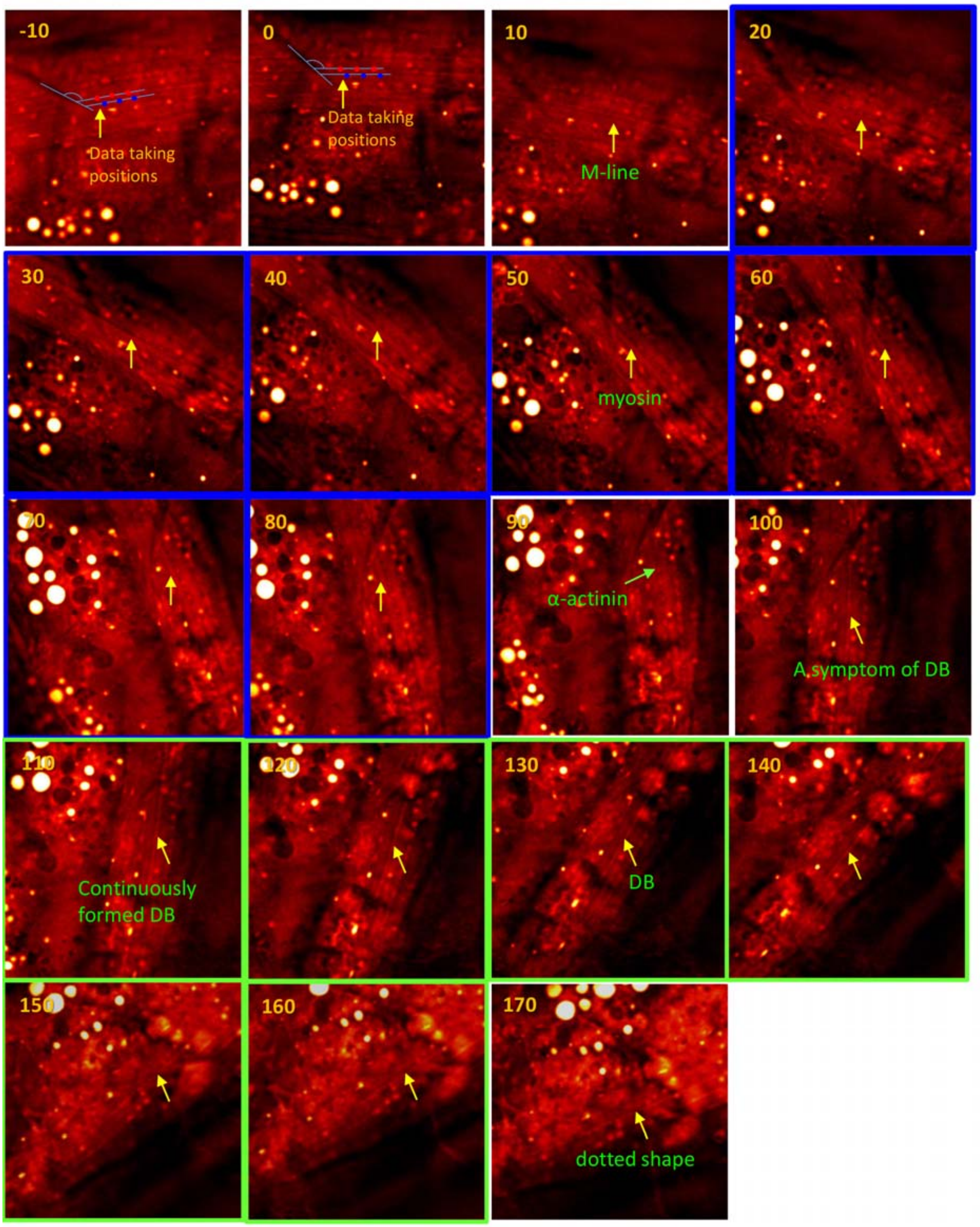

Figure S9. Polarization-dependent spCARS images of *C. elegans*. Images surrounded by a blue square are myosin-rich; those surrounded by a green square are DB-rich.

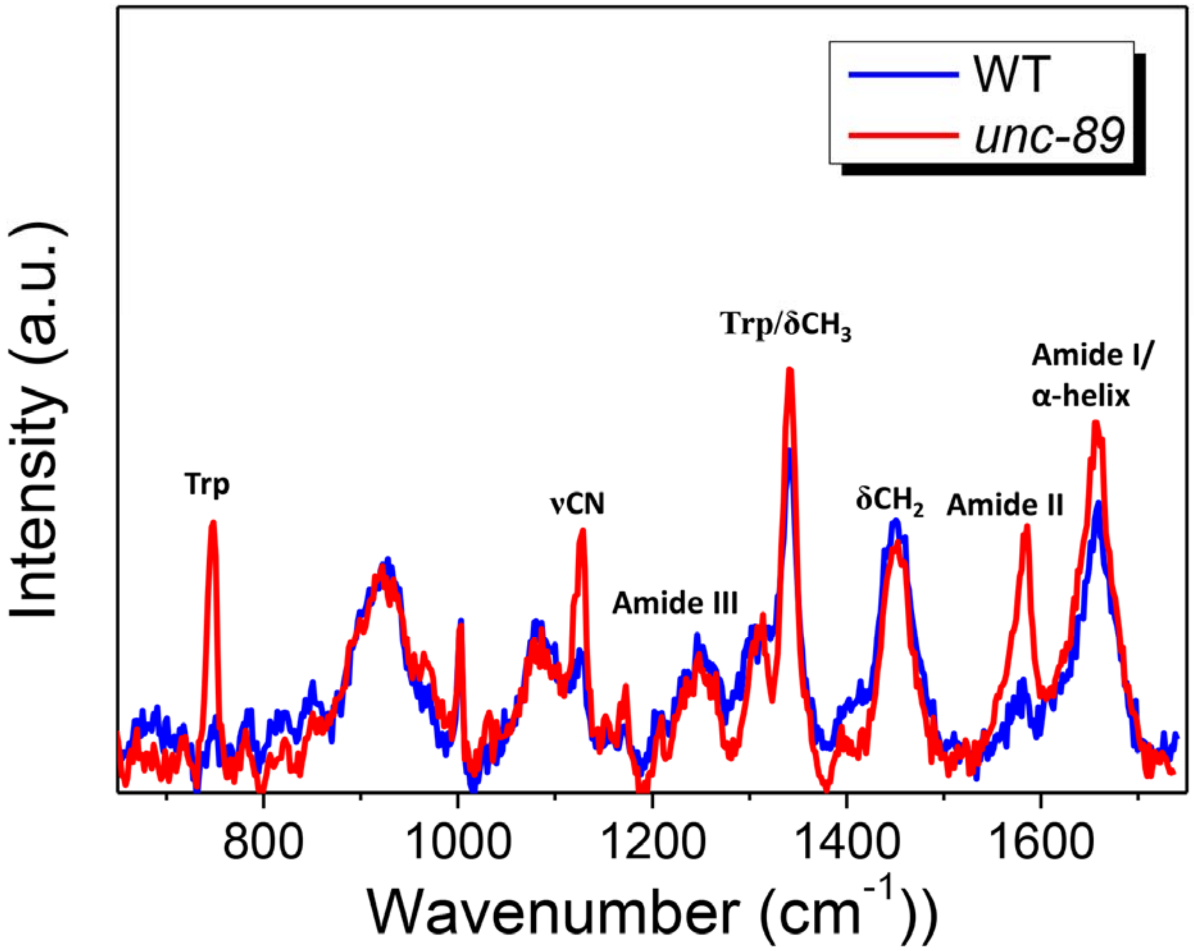

Figure S10. Fingerprint region of the spontaneous Raman spectra for TBs of WT (blue) and *unc-89* (red) worms.

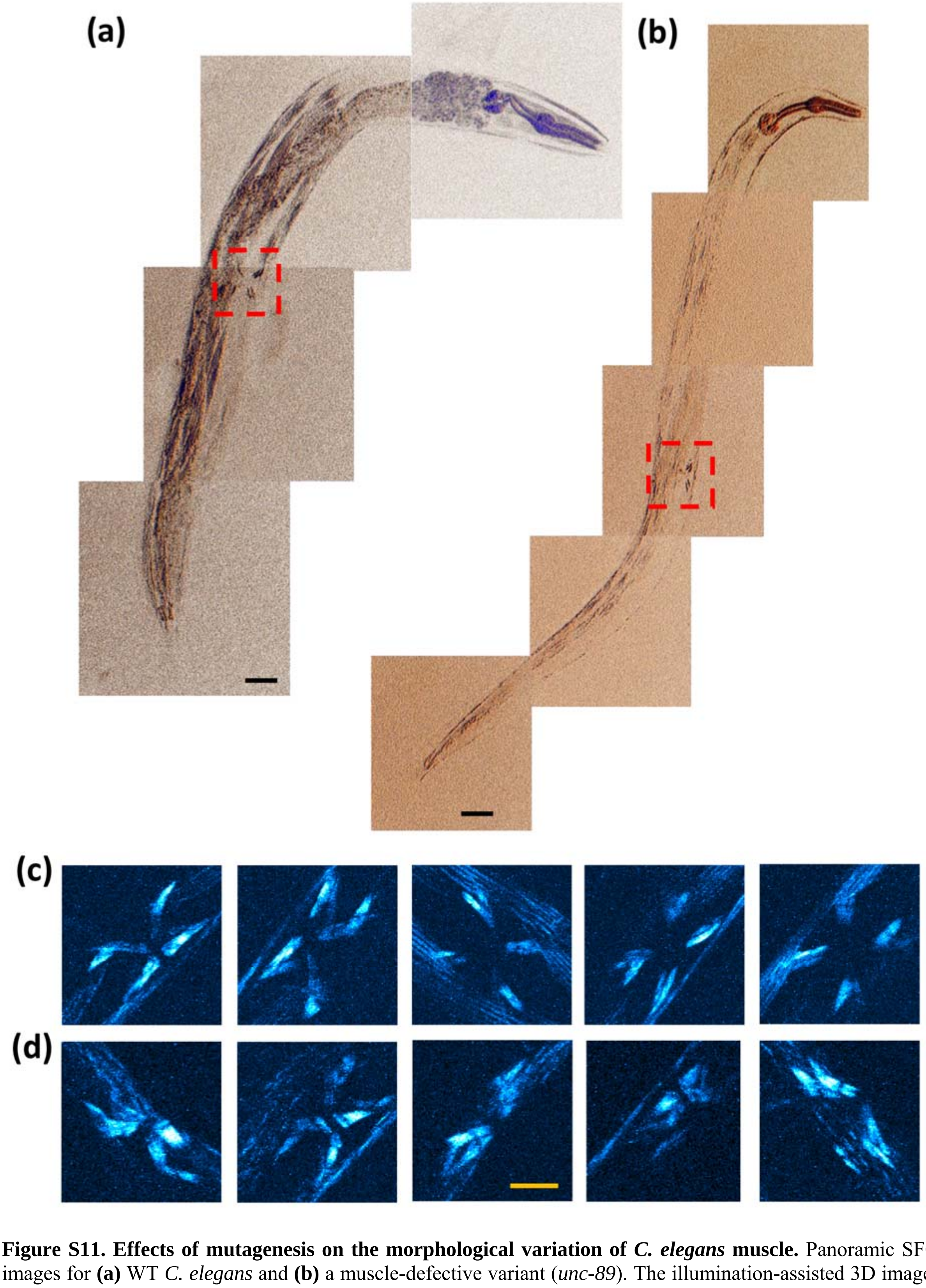

Figure S11. Effects of mutagenesis on the morphological variation of *C. elegans* muscle. Panoramic SFG images for **(a)** WT *C. elegans* and **(b)** a muscle-defective variant (*unc-89*). The illumination-assisted 3D images were created using the 3D volume viewer tool of the ImageJ software. **(c)** and **(d)** show SFG images of the vulva of WT and *unc-89* strains, respectively. The scale bars represent 20 μm.

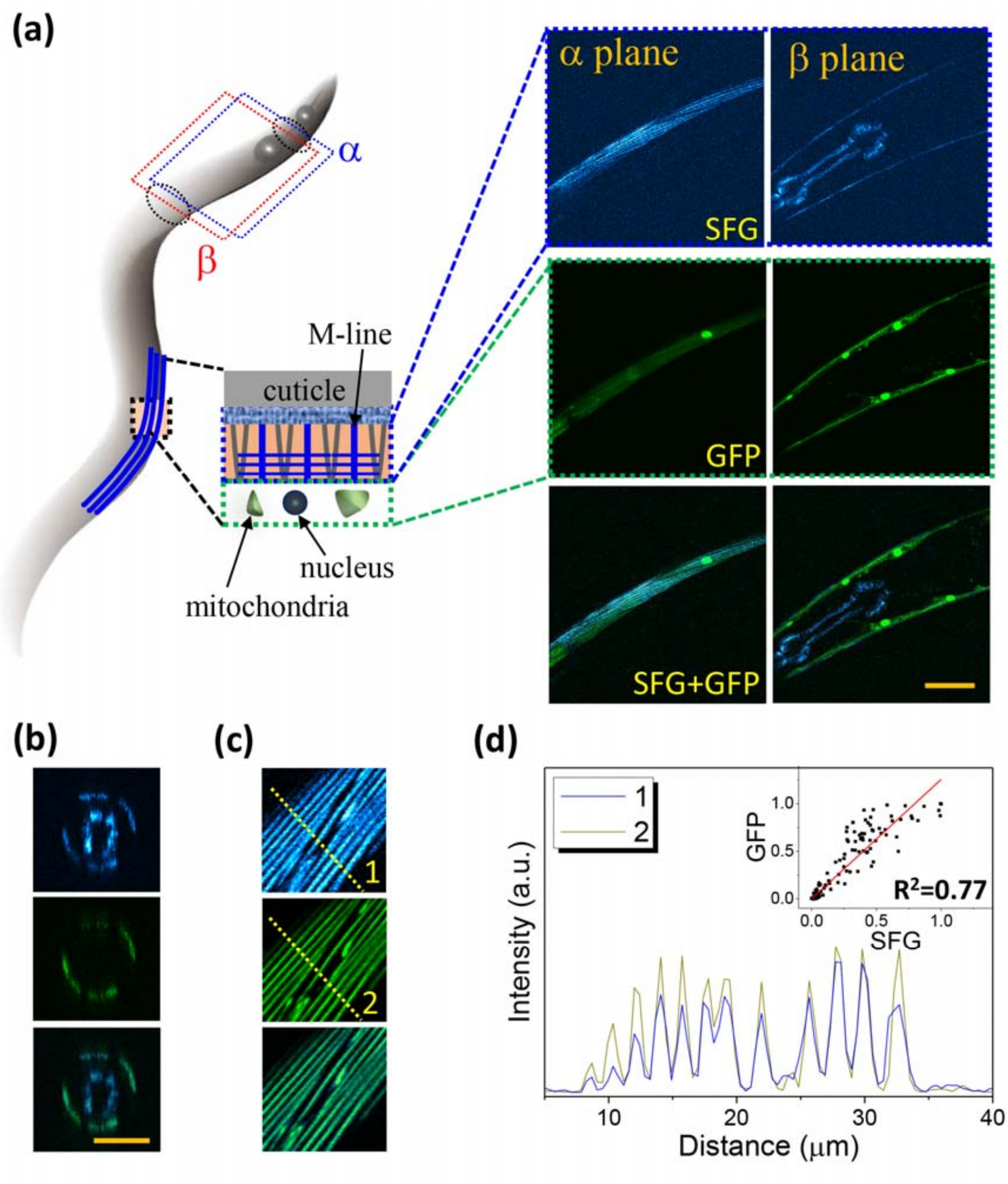

Figure S12. Comparison between mitochondria-labeled GFP and label-free SFG images. (a) Schematic depicting the structure of WT *C. elegans* muscle. The blue dashed (α) rectangular plane produced a topological view of the body wall muscle while the red dashed (β) plane selectively captured a section including the pharyngeal muscle as well as the outer part of the body wall muscle. The mitochondria are clearly seen as dots in the two-photon excited luminescence GFP images. **(b)** *z*-directional cross-sections of the TB and **(c)** zoomed-in topological views of body wall muscle for the RW1596 strain, displayed in SFG, GFP, and overlay images (from top to bottom). **(d)** Normalized intensity line profiles for SFG (1) and GFP (2), showing that the correlation factor R^2 is ~0.77. The scale bars represent 50 μm.

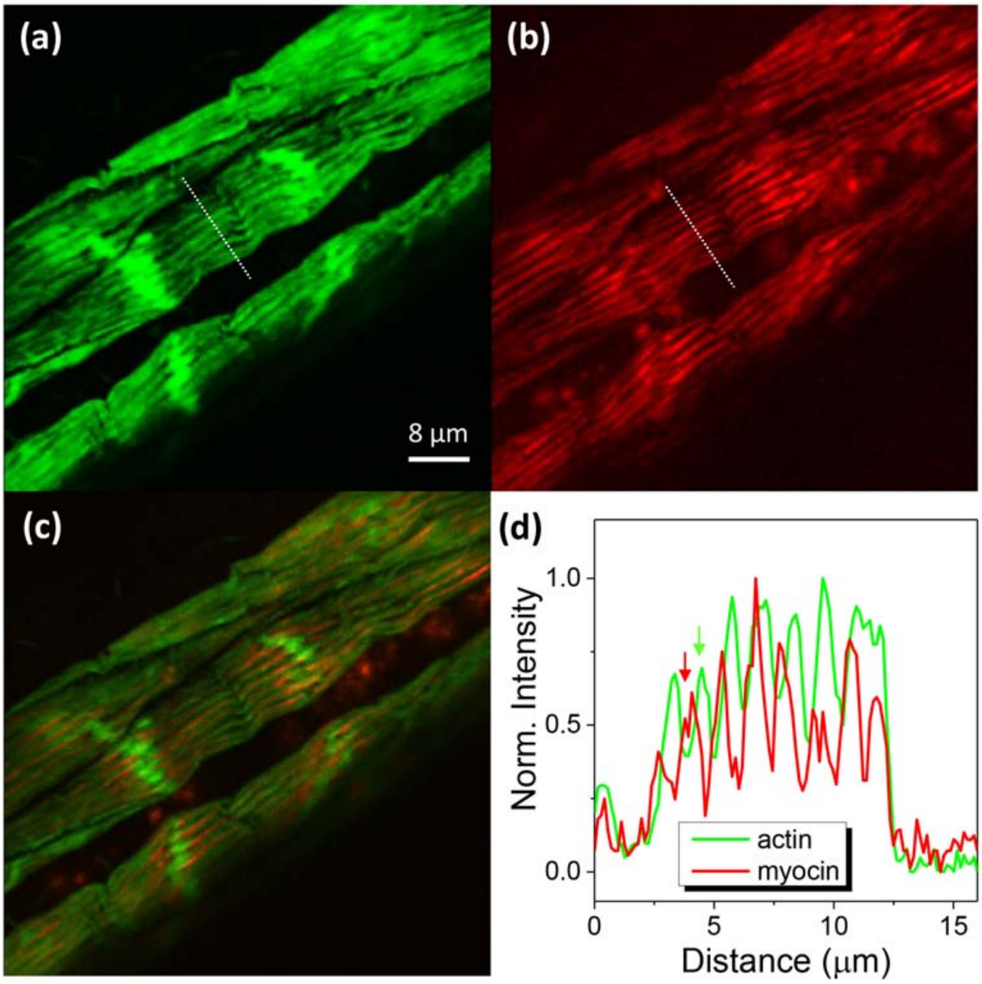

Figure S13. Identification of myosin by comparing SFG and actin-labeled GFP signals. (a) TPEF image of body wall muscle of WT *C. elegans* stained with fluorescein phalloidin (F432, Thermofisher Scientific). **(b)** SFG image taken simultaneously with (a). The image was colored red (instead of cyan) for better comparison when overlaying. **(c)** Overlay image of (a) and (b). **(d)** Line profiles from the white dotted lines in (b) and (c). There is a noticeable mismatch between the actin-rich (green) and myosin-rich (red) sites.

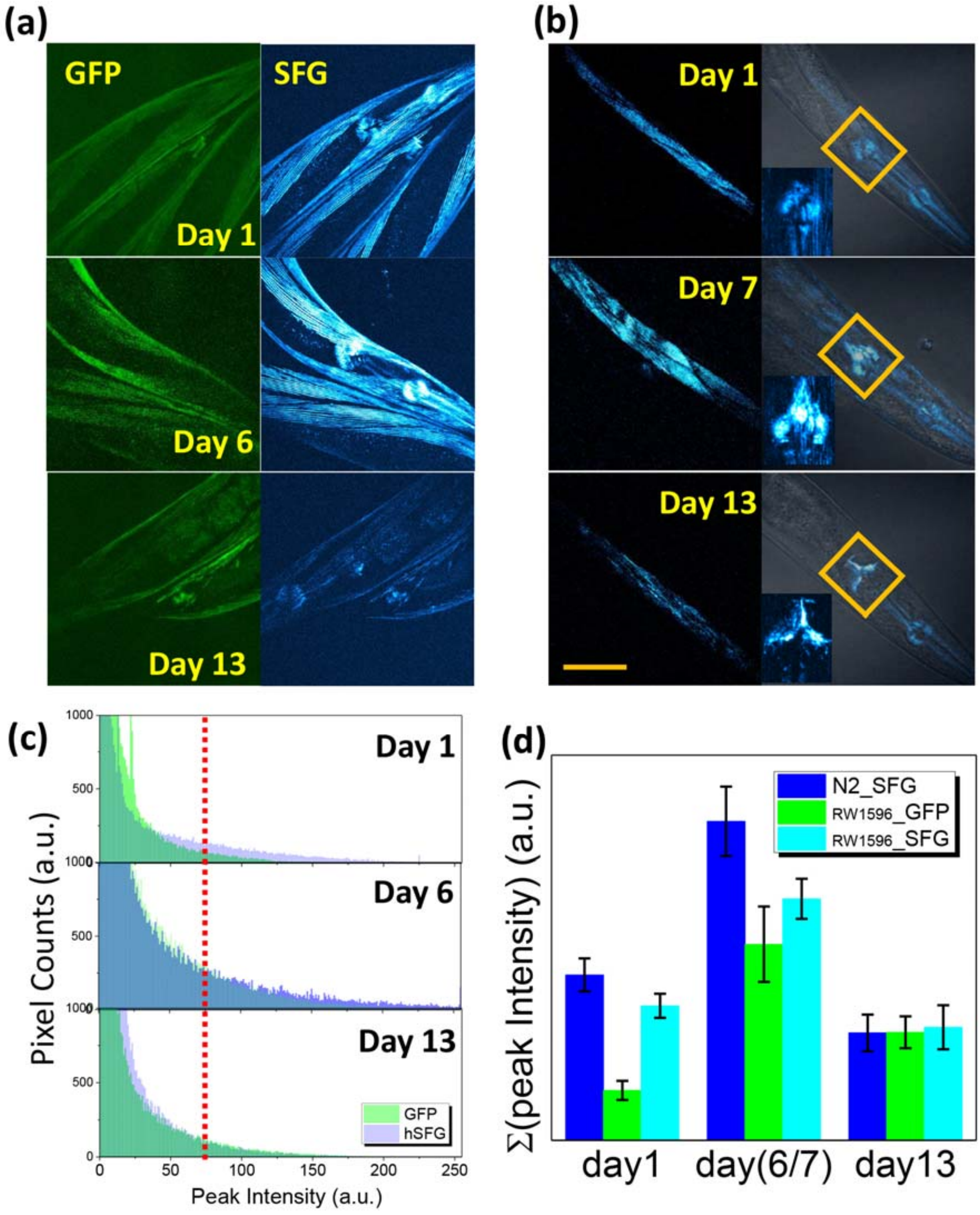

Figure S14. Aging-dependent SFG generation calibrated with a GFP-labeled sample. (a) GFP (left panel) and SFG (right panel) images of 1-, 6-, and 13-day old RW1596 strain samples. **(b)** SFG images (left panel) of a 1-, 7-, and 13-day old WT worm. Overlay images (transmission + SFG, right panel) produced by taking the point-by-point intensity maximum from image stacks of *z*-directional sections. The insets show selected SFG images of pharyngeal TBs for each age. **(c)** Intensity histograms for the GFP/SFG signals from 1-, 6- and 13-day old RW1596 samples. **(d)** Comparison of the total peak intensity of the SFG signal from WT/RW1596 samples and that of the GFP signal from RW1596 samples.

Table S1. Multiple peak deconvolution of the phase-retrieved spCARS spectra as a function of the central wavenumber, the FWHM, and the peak area in Figure 1e.

	**Position (1) and (2)**		**Position (3)**	
**Wavenumber (cm^{-1})**	**FWHM (cm^{-1})**	**Area (a.u.)**	**FWHM (cm^{-1})**	**Area (a.u.)**
2849	11.0	1.0	11.4	0.5
2859	17.0	2.4	18.0	7.4
2881	32.1	3.0	32.0	17.1
2903	35.2	30.2	39.1	35.9
2932	44.8	50.1	45.0	45.1
2973	44.7	36.1	44.8	30.1
3009	20.1	3.8	21.0	6.8
3035	18.0	2.9	18.0	3.5

Table S2. Multiple peak deconvolution of the phase-retrieved spCARS spectra as a function of the central wavenumber, the FWHM, and the peak area in Figures 4c and 4d.

	**Figure 4c**		**Figure 4d**	
**Wavenumber (cm^{-1})**	**FWHM (cm^{-1})**	**Area (a.u.)**	**FWHM (cm^{-1})**	**Area (a.u.)**
2849	11.4	1.7	11.4	0.6
2859	17.1	5.8	17.1	2.9
2881	32.0	18.7	32.0	16.5
2903	38.8	17.8	38.8	12.2
2932	60.0	75.0	64.0	85.1
2973	42.1	18.9	50.0	20.1
3009	35.1	2.1	30.0	7.0
3035	37.6	1.2	40.0	12.0

Table S3. Multiple peak deconvolution of the phase-retrieved spCARS and spontaneous Raman spectra as a function of the central wavenumber, the FWHM, and the peak area in Figures 5g–j.

	Figure 5g		Figure 5h		Figure 5i		Figure 5j	
Wavenumber (cm^{-1})	FWHM (cm^{-1})	Area (a.u.)	FWHM (cm^{-1})	Area (a.u.)	FWHM (cm^{-1})	Area (a.u.)	FWHM (cm^{-1})	Area (a.u.)
2849	11.4	1.6	11.4	2.4	11.4	2.2	11.0	1.1
2859	17.0	5.0	17.1	6.0	17.0	3.6	17.0	2.6
2881	32.0	17.4	32.0	19.6	32.0	13.3	32.0	10.7
2903	38.8	22.3	38.8	24.4	38.0	16.4	40.0	15.1
2932	53.3	63.0	53.0	56.4	34.5	28	45.0	33.0
2973	40.0	22.0	45.0	18.4	36.1	12	50.1	17.2
3009	20.4	3.1	35.0	10.1	20.0	1.2	22.0	2.4
3035	17.9	2.1	34.9	9.8	17.9	0.9	32.0	1.2
3078	-	-	34.8	7.1	-	-	42.0	14.8

Supporting references

[1] D. Lee, D.-E. Jeong, H.G. Son, Y. Yamaoka, H. Kim, K. Seo, A.A. Khan, T.-Y. Roh, D.W. Moon, Y. Lee, S.-J.V. Lee, SREBP and MDT-15 protect *C. elegans* from glucose-induced accelerated aging by preventing accumulation of saturated fat. Genes & Dev. 29, 2490-2503 (2015).

[2] D. Fu, G. Holtom, C. Freudiger, X. Zhang, X. Sunney Xie, Hyperspectral Imaging with Stimulated Raman Scattering by Chirped Femtosecond Lasers. J. Phys. Chem. B 117, 4634-4640 (2013).

[3] B. Liu, H.J. Lee, D. Zhang, C.-S. Liao, N. Ji, Y. Xia, J.-X. Cheng, Label-free spectroscopic detection of membrane potential using stimulated Raman scattering. Appl. Phy. Lett. 106, 173704 (2015).

[4] S.-H. Kim, E.-S. Lee, J.Y. Lee, E.S. Lee, B.-S. Lee, J.E. Park, D.W. Moon, Multiplex coherent anti-Stokes Raman spectroscopy images intact atheromatous lesions and concomitantly identifies distinct chemical profiles of atherosclerotic lipids. Circ. Res. 106, 1332-1341 (2010).

[5] R.S. Lim, J.L. Suhalim, S. Miyazaki-Anzai, M. Miyazaki, M. Levi, E.O. Potma, B.J. Tromberg, Identification of cholesterol crystals in plaques of atherosclerotic mice using hyperspectral CARS imaging. J. Lipid Res. 52, 2177-2186 (2011).

[6] C. H. Camp Jr., Y. J. Lee, M. T. Cicerone, Quantitative, comparable coherent anti-Stokes Raman scattering (CARS) spectroscopy: correcting errors in phase retrieval. J. Raman. Spectrosc. 47, 408–415 (2016).

[7] S. Bresson, D. Bormann, B. Khelifa, Raman studies of the C–H stretching modes in various cholesteryl alkanoates. Vib. Spec. 16, 163-171 (1998).

[8] I. Rocha-Mendoza, D.R. Yankelevich, M. Wang, K.M. Reiser, C.W. Frank, Sum Frequency Vibrational Spectroscopy: The Molecular Origins of the Optical Second-Order Nonlinearity of Collagen. Biophys. J. 93, 4433-4444 (2007).

[9] R. Eckela, H. Huoa, H.-W. Guanb, X. Hub, X. Chea, W.-D. Huanga, Characteristic infrared spectroscopic patterns in the protein bands of human breast cancer tissue. Vib. Spectrosc. 27, 165-173 (2001).
